# A genome-wide association study of the racing performance traits in Yili horses based on Blink and FarmCPU models

**DOI:** 10.1038/s41598-024-79014-w

**Published:** 2024-11-12

**Authors:** Chuankun Wang, Yaqi Zeng, Jianwen Wang, Tongliang Wang, Xueyan Li, Zhehong Shen, Jun Meng, Xinkui Yao

**Affiliations:** 1 College of Animal Science, Xinjiang Agricultural University, Urumqi, 830052 China; 2 Xinjiang Key Laboratory of Horse Breeding and Exercise Physiology, Urumqi, 830052 China

**Keywords:** Yili horses, GWAS, Racing performance, Speed, Ranking score, Genetics, Molecular biology

## Abstract

Racing performance traits are the main indicators for evaluating the performance and value of sport horses. The aim of this study was to identify the key genes for racing performance traits in Yili horses by performing a genome-wide association study (GWAS). Breeding values for racing performance traits were calculated for Yili horses (*n* = 827) using an animal model. Genome-wide association analysis of racing performance traits in horses (*n* = 236) was carried out using the Blink, and FarmCPU models in GAPIT software, and genes within the significant regions were functionally annotated. The results of GWAS showed that a total of 24 significant SNP markers (*P* < 6.05 × 10^− 9^) and 22 suggestive SNP markers (*P* < 1.21 × 10^− 7^) were identified. Among them, the Blink associated 16 significant SNP loci and FarmCPU associated 12 significant SNP loci. A total of 127 candidate genes (50 significant) were annotated. Among these, *CNTN6* (motor coordination), *NIPA1* (neuronal development), and *DCC* (dopamine pathway maturation) may be the main candidate genes affecting speed traits. *SHANK2* (neuronal synaptic regulation), *ISCA1* (mitochondrial protein assembly), and *KCNIP4* (neuronal excitability) may be the main candidate genes affecting ranking score traits. A common locus (ECA1: 22698579) was significantly associated with racing performance traits, and the function of the genes at this locus needs to be studied in depth. These findings will provide new insights into the detection and selection of genetic variants for racing performance and will help to accelerate the genetic improvement of Yili horses.

**Supplementary Information:**

The online version contains supplementary material available at 10.1038/s41598-024-79014-w.

## Introduction

Regional preferences for certain traits have resulted in phenotypic variation, which may result from adaptations to the local racing ecosystem^1^. Although racing traits are complex, selecting racehorses with traits common to winners in a given environment for breeding can increase the probability of genetic variation in those traits in the offspring. Over time, systematic selection can optimize the population’s genome^2^. Therefore, knowledge of association between traits and influential genotypes will help breeders produce healthier more sustainable, and better-performing horses^3^.

Equine research and breeding have encountered major changes due to the rapid development of molecular genetics technology^4,5^. Genome-wide association studies (GWAS) have been successfully deployed to identify quantitative trait loci (QTLs) for complex traits using relatively modest sample sizes^6–8^. Today, there are 302 horse traits listed on the Online Mendelian Inheritance in Animals (OMIA) website, while the HorseQTLdb lists 2216 QTLs representing 61 traits. A total of 431 QTLs were identified as being related to racing ability, gait, and jumping ability of horses. These key genetic markers offer the possibility of applications for genetic testing and selection in horses^9–12^.

The thoroughbred, developed relatively quickly over the last three centuries through crossbreeding of local British mares with Middle Eastern stallions, has become the world’s most successful racehorse. Most thoroughbreds compete in races over much shorter distances (1000–3200 m) and are bred for both speed and stamina^13^. Fewer founders, large populations, stronger selection pressures, and lower genetic diversity make the racing traits and genomic structure of Thoroughbreds suitable for study^13–15^. A wealth of genetic information related to racing distance^16^, speed^3^, rankings^17^, and longevity of participation^18^ have been reported. Today, thoroughbreds are widely used to improve the racing performance of other horse breeds.

The Yili horse, originating from the Yili Kazakh Autonomous Prefecture in the Xinjiang Uygur Autonomous Region of China, was developed during the last century by crossing native Kazakh mares with stallions of Orlov, Budyonny, and Don River breeds^19^. To meet different production needs, there are several phenotypically and genetically distinct subgroups of Yili horses that are used for meat, milk, and racing. The Yili racehorse group includes several types of galloping, trotting and pacing. The gallop type was developed through the crossbreeding of Yili mares with thoroughbred stallions, to combine their best qualities^20^. This group has become one of the most influential horse racing groups in China due to the standardized holding of racing events and breeder preferences. Horse racing is held annually during the Xinjiang Tianma Cultural and Tourism Festival, where young (2–3-year-old) and adult Yili horses compete in races over short, medium, and long distances (1000–5000 m) and are bred for both speed and stamina attributes.

In recent years, the racing performance of the Yili horse has been improving from the crossbreeding with thoroughbred stallions and the strong selection for racing capability. The focus of our team’s research has gradually shifted from analyzing physiological and biochemical indicators in racehorses to genomics^21–23^. Previous studies found some key polymorphisms in the *MSTN*, *GH*, *DMRT3*, *COMT* genes and sought to determine the relationship between these genes and body size, gait, racing performance and cardiac function^24–27^. We obtained some inferential conclusions but lacked large data samples to demonstrate significant effects. Consequently, in the present study, we hypothesized that the enhancement of racing performance in the racing population of Yili horses is genetically influenced by thoroughbred stallions and that there are some genes or genomic regions associated with race performance traits. Therefore, we first analyzed the phenotypic data of Yili racehorses (gallop type), using the breeding values of race performance traits of Yili horses and thoroughbred stallions as the phenotypic data. Genotype data were obtained through 5x and 10x whole-genome resequencing. Lastly, GWAS technology was used to identify genetic markers that were closely related to racing performance, which provides a reference for the selective breeding of Yili horses.

## Materials and methods

### Experimental animals and phenotypic data

The studied populations consisted of Yili horse (gallop types, *n* = 827), and thoroughbreds (studs, *n* = 134) from Xinjiang Uygur Autonomous Region, Northwest China. A total of 2576 flat racing records and 12,546 g-pedigree data entries from 827 Yili horses for 9 years (2015 to 2023) were used to estimate breeding values for racing performance traits. Based on the tracing of the horse information, a total of 212 Yili horses (118 stallions and 94 mares) with qualified race records (*n* ≥ 6) and 41 Thoroughbreds (studs) with progeny numbers (*n* ≥ 50) were selected for DNA re-sequencing. The sequencing depth was 5X and 10X, respectively.

Racing performance data collection was carried out from February to November each year using a standardized 2000 m sand track and electronic timing system. The study traits were as follows: (1) Average speed (AS) was the average speed of the horse completing the race. Ranking score (RS) was the sum of the ranking score of the horses in the competition and the time gap between the horse and the leading horse using the following formula: RS = ( K - K_X_ )*100 + ( RT_X_ - RT_F_ ), where K is the total number of horses in the race; K_X_ is the ranking of the x horse; RT_X_ is the race time of the x horse, and RT_F_ is the race time of the winning horse.

### Estimated breeding values

The significance (*P* < 0.05) of the fixed effects of racing performance traits in Yili horses was tested using the GLM process (SAS 8.1). We considered that the age of racing, racing distance, year of birth, gender of horse, month of racing, and level of racing to be fixed effects, and individual additive genetic effects as random effects. The results of descriptive statistics (Tables S1) and fixed effects significance test (Tables S3-9) are provided in the Supplementary Material.

The estimates of genetic parameters and breeding values (EBVs) for the speed and ranking score traits were determined using the single trait repeatability model from the DMU software. The genetic and phenotypic correlations were determined using multi-trait animal models (DMUs), and the standard errors (SEs) of genetic and phenotypic correlations were estimated using the method of Klei and Tsuruta^28^. The single trait breeding values + residuals as phenotypic values^29^ were used to carry out the GWAS. Thesingle trait repeatability model equation is as follows:

$${\mathbf{Y}}\, = \,{\mathbf{X\upbeta }}\, + \,{\mathbf{Za}}\, + \,{\mathbf{Wpe}}\, + \,{\mathbf{e}}.$$

where **Y** is the vector of observations, **β** is the vector of fixed effects, **a** is the vector of additive genetic effects, **pe** is the vector of permanent environmental factors of individuals for speed and ranking score trait, and **e** is the vector of residuals. **X** and **Z** are the incidence matrices corresponding to fixed and additive effects, respectively, and **W** is the permanent environmental effect incidence matrix.

### DNA resequencing data

Blood samples from the Yili horses (*n* = 212) and Thoroughbreds (*n* = 41) were collected with the owner’s consent between 2021 and 2023. DNA was extracted from the blood samples using a GenoPrep animal tissue DNA extraction kit with magnetic beads (Mix-V4.0, Boridi, Hebei, China). The DNA fragments were end-repaired, A-tailed, adaptor-ligated, and amplified using the Dongshengxing ETC821 bioanalyzer (Dongsheng, Jiangsu, China, USA). QC of DNA samples was performed by agarose gel electrophoresis to determine the extent of DNA degradation and the presence of heterobands, RNA, and protein contamination; Qubit 2.0 fluorometry was used to measure DNA concentration.

DNA resequencing libraries were constructed using the GenoBaits DNA library prep kit for ILM (BioVision, San Francisco, CA, USA) on quality-checked DNA. Sequencing was performed using an MGI-2000/MGI-T7 sequencing platform (Shenzhen UW Smart Technology, Shenzhen, China). In this study, each base sequence was quality-checked (-w 4 -q 20 -n 2 -u 30) using Fastp software (ver. 0.20.0)^30^. The paired-end sequences were localized to the equine reference genome (*Equus caballus* 3.0) using BWA (ver. 0.7.17)^31^. Variant detection was performed using the HaplotypeCaller module of GATK (ver. 4.0.4.0)^32^.

### Variant site filtering

**Fig. 1:**
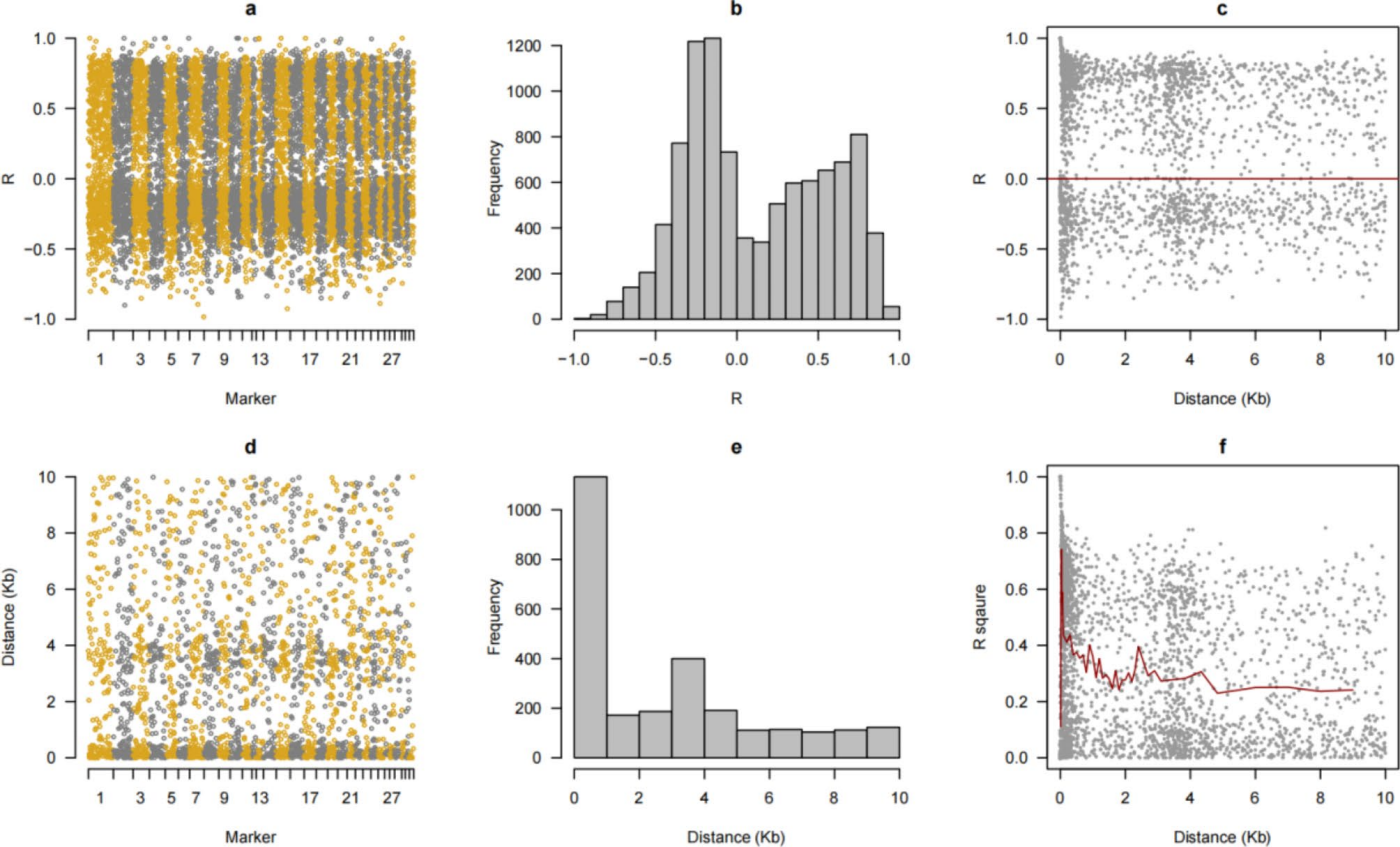
Genotype analysis including R^2^ (**a**, **b** and **c**) and distance (**d**, **e** and **f**).

PLINK software^33^ was used for QC of the sequencing data with the following criteria: minor allele frequency (MAF) < 5%, individual detection rate < 95%, SNP missing rate < 90%, and Hardy-Weinberg equilibrium *P* value > 10^− 4^. Sequencing yielded 22,039,238 SNPs, and 10,741,200 SNPs were obtained for genotypic analysis after data quality control. We calculated marker intervals and linkage disequilibrium (LD) to estimate R^2^ for all markers and plotted the marker distribution (Fig. 1). The frequency, MAF and heterozygosity values are shown in the supplementary material (Figure S1-2).

### Population structure

**Fig. 2:**
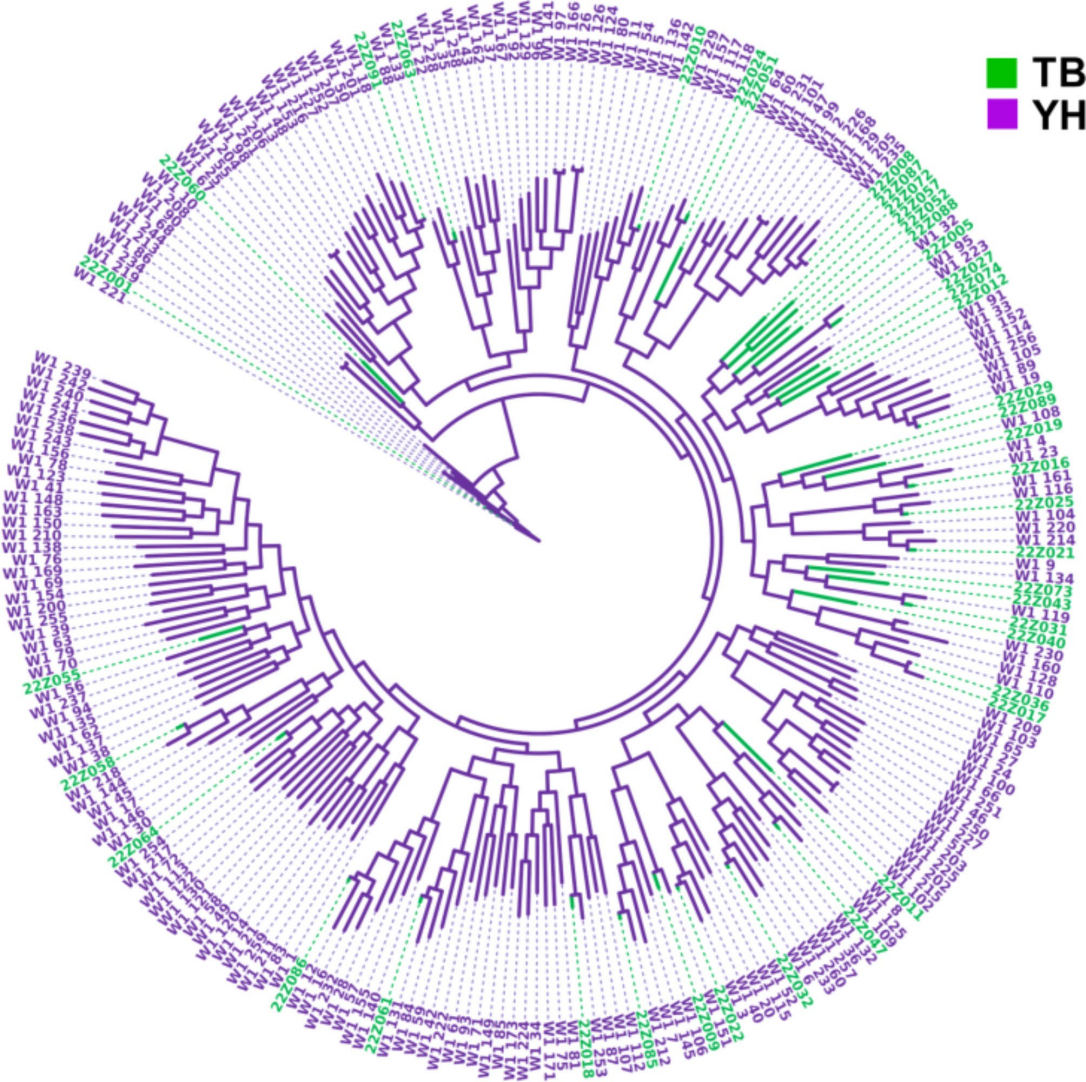
Phylogenetic evolutionary trees of the samples (TB, thoroughbreds; YH, Yili horses).

Based on the SNP markers obtained by quality control, Phylogenetic evolutionary trees were constructed using the IQ-TREE 2 and iTOL^34,35^ (Fig. 2).

### Genome-wide association study

To assess the potential associations between genetic loci and traits at the genomic level, genome-wide association studies (GWASs) were performed using GAPIT (ver 3)^36^, which integrates multiple algorithms for association analysis and ensures that plausible associations of loci are screened by multiple methods that corroborate each other. The GWAS models used in this study include Bayesian information and linkage disequilibrium iterative nested keyway (Blink)^37^, and fixed stochastic cyclic probabilistic uniform (FarmCPU)^38^. The GCTA software (ver 1.92.4) was used to determine population stratification and relatedness in Yili horses, and the results were used as random effects in a GWAS.

Genome-wide association analysis significance thresholds (6.05 × 10^− 9^) and suggestive significance thresholds (1.21 × 10^− 7^) were determined using a value of 0.05/n, 1/n, where *n* (8235197) is the number of independent SNPs computed using the genetic type 1 error calculator (v.0.2; https://pmglab.top/gec/#/)^39,40^.

The proportion of variance explained (PVE): was calculated as follows^41^:

$${\mathbf{PVE}}=\:\:\frac{2{\varvec{\beta\:}}^{2}\varvec{M}\varvec{A}\varvec{F}(1-\varvec{M}\varvec{A}\varvec{F})}{2{\varvec{\beta\:}}^{2}\varvec{M}\varvec{A}\varvec{F}(1-\varvec{M}\varvec{A}\varvec{F})+{\left(\varvec{s}\varvec{e}\right(\varvec{\beta\:}\left)\right)}^{2}2\varvec{N}\varvec{M}\varvec{A}\varvec{F}(1-\varvec{M}\varvec{A}\varvec{F})}$$

where $\:\varvec{\beta\:}$ is the effect of SNP markers, **MAF** is the frequency of SNP marker minor alleles, $\:\varvec{s}\varvec{e}\left(\varvec{\beta\:}\right)\:$is the standard error of the effect of SNP markers, and **N** is the number of samples analyzed by GWAS.

### Gene function annotation

The, reference genome (*EquCab3.0*) of the horse, *Equus caballus*, was downloaded from the National Center for Biotechnology Information (NCBI) site, and the 100 kb region before and after the significant locus was annotated by ANNOVAR^42^. GO and KEGG enrichment analyses were performed using DAVID (https://david.ncifcrf.gov/summary.jsp)^43–46^. The animal QTLdb NR database was used to find significant loci and gene functions, and the database was also used for functional gene mining of associated intervals^47^.

### Data availability statement

Sequences are available from GSA with the BioProject accession number PRJCA023926 (https://www.cncb.ac.cn/).

## Results

### Descriptive statistics

**Fig. 3:**
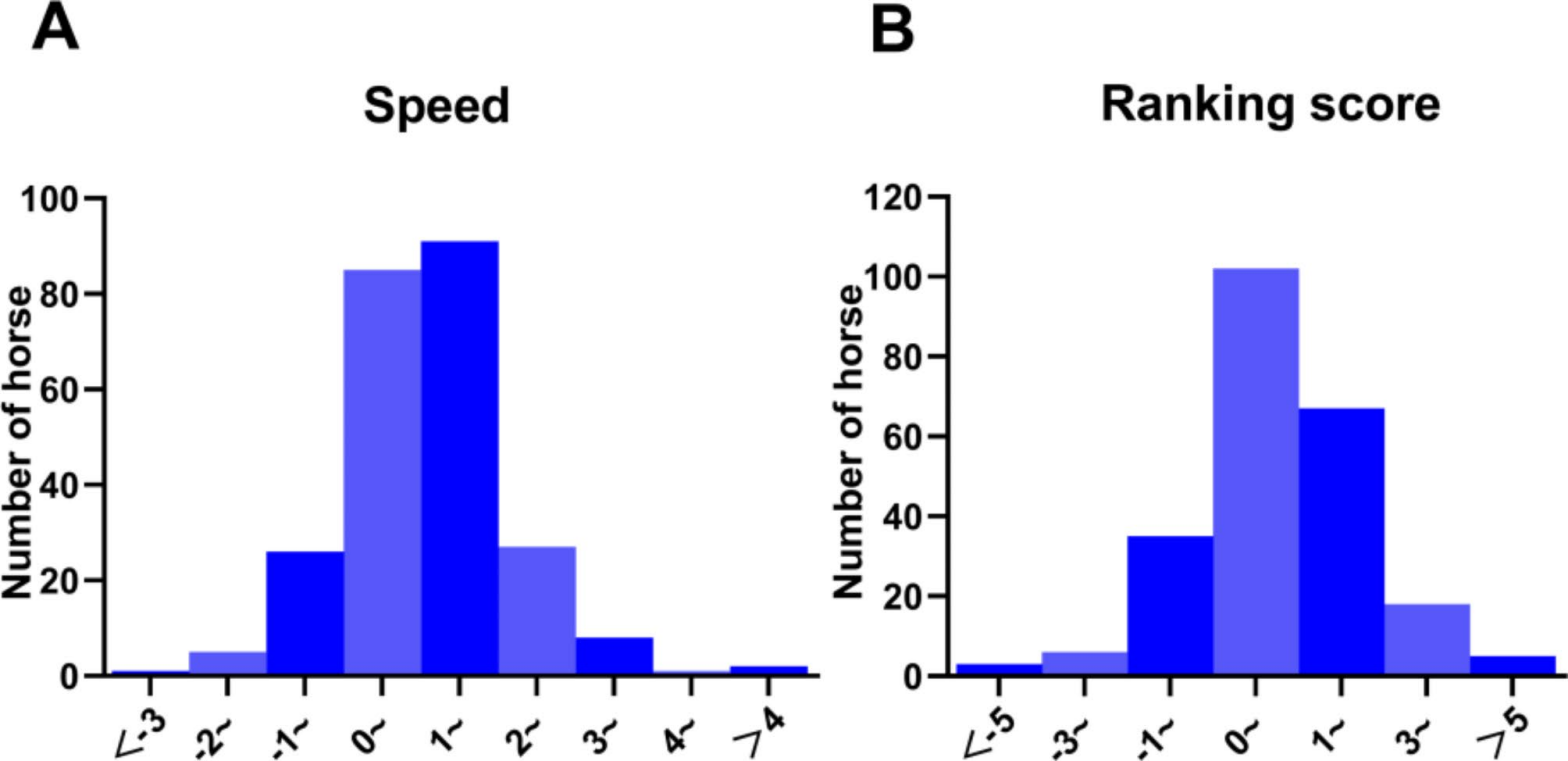
Frequency distribution of estimated breeding values (EBVs) for speed (**A**) and ranking score (**B**) traits in Yili horses.

In this study, a total of 2576 speed and ranking score records were used as data for genetic parameter estimation. The results of variance component estimation are given in Table 1, which shows that speed and ranking score had moderate heritability (0.347, 0.156). We also found a highly significant positive genetic correlation (0.920) and phenotypic correlation (0.735), which is shown in the Supplementary Material (Tables S2). The frequency distribution of data of EBVs for speed and ranking score traits were normally distributed (Fig. 3).

**Table 1:** Estimates of variance components and heritability for racing performance traits.

Traits	Number	$\sigma _{{\text{a}}}^{{\text{2}}}$	$\sigma _{{{\text{pe}}}}^{{\text{2}}}$	$\sigma _{{\text{e}}}^{{\text{2}}}$	$\sigma _{{\text{e}}}^{{\text{2}}}$	pe^2^	h^2^(SE)	re
AS	2576	0.249	0.185	0.284	0.718	0.257	0.347(0.020)	0.604
RS	2576	60.407	71.298	254.334	386.039	0.184	0.156(0.026)	0.341

AS, average speed; RS, ranking score; σ_a_^2^, additive genetic variance; σ_pe_^2^, permanent environmental variance; σ_e_^2^, residual variance; σ_p_^2^, phenotypic variance; pe^2^, proportion of phenotypic variance explained by permanent environmental effects; ℎ^2^, heritability; SE, standard error; re, repeatability.

### Resequencing of Yili horses

**Fig. 4:**
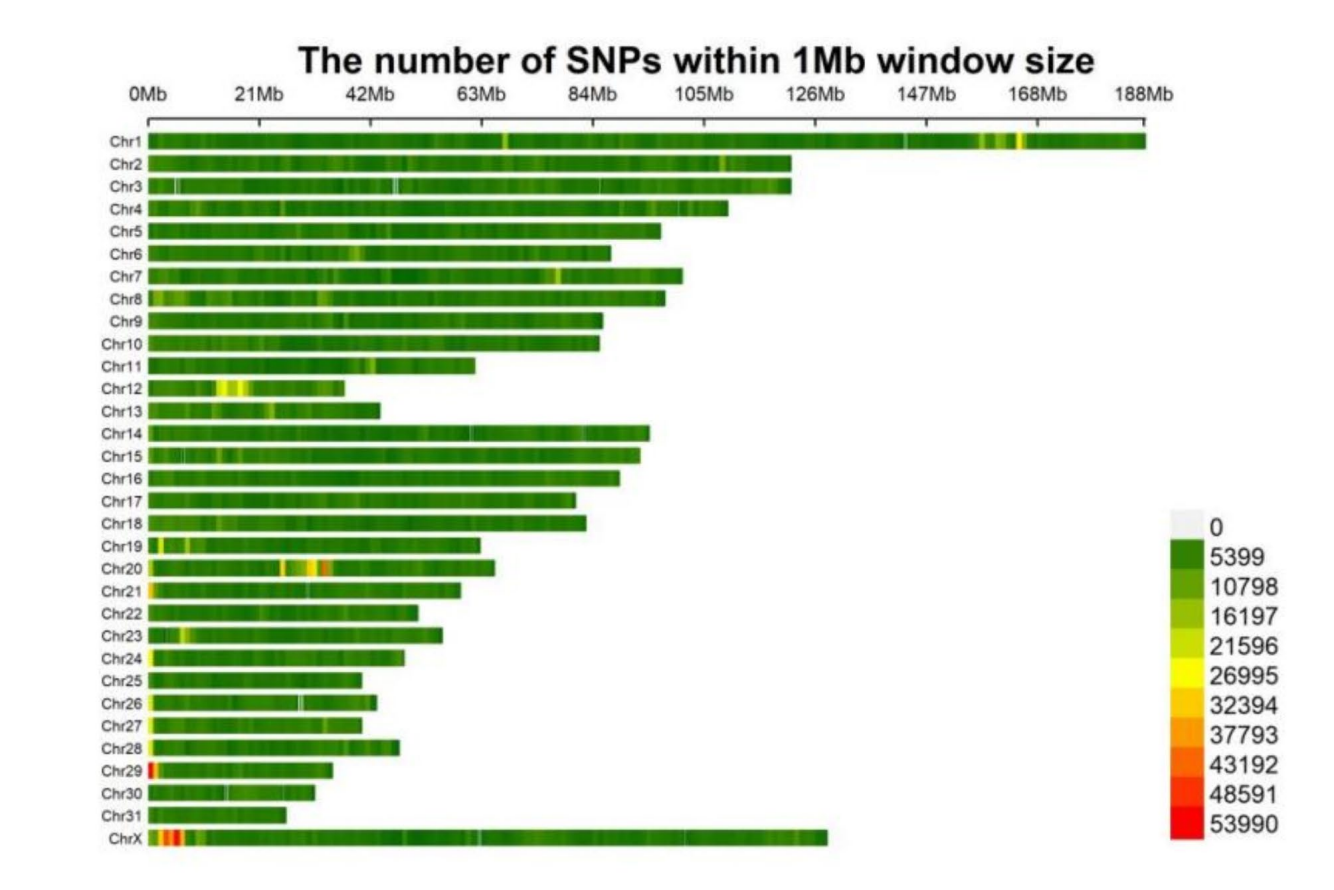
Density distribution of re-sequenced SNP sites in Yili horses.

**Fig. 5:**
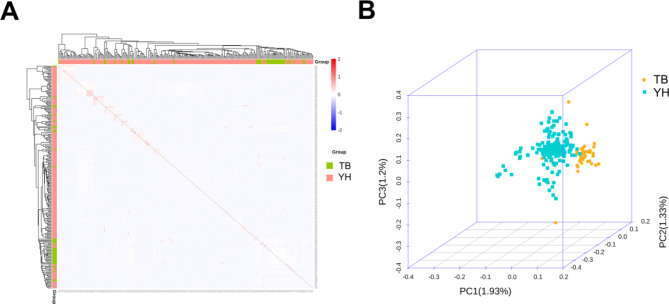
Kinship (**A**) and population stratification (**B**) of sample groups (TB, thoroughbreds; YH, Yili horses).

The sequence alignment to the reference genome was 99.34%, and the average depth of sequencing was 7.59X, with 71.58% at 5X coverage, and 21.30% at 10X coverage (see supplementary material Table S10-11). The results of the genome testing are shown in Fig. 4. The sequencing data were evenly distributed throughout the genome, with good sequencing randomness, and the SNPs had a high-density distribution on *ECA 20*, *29*, and *X*. Kinship matrices as random effects and principal component analysis (PCA) as covariates were added to the GWAS analysis model. (Fig. 5). Additional pca results are included in supplementary Material (Figure S3-5). According to the Kinship, PCA, and evolutionary relationship between populations, we finally selected 212 Yili horses and 24 thoroughbreds for GWAS analysis.

### Association analysis

**Fig. 6:**
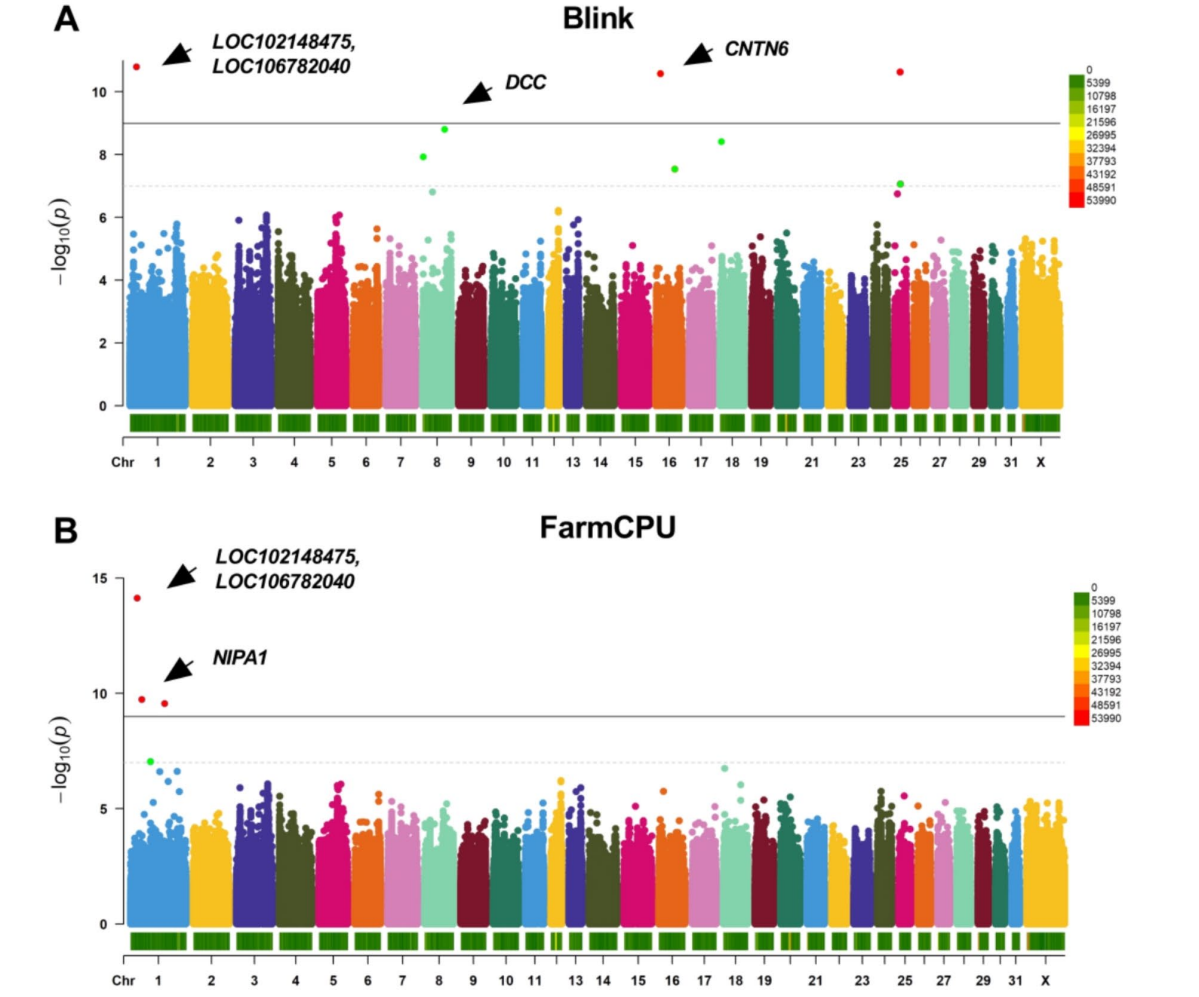
Manhattan plots of speed trait base on Blink (**A**) and FarmCPU (**B**). The values in the upper right corner of the image indicate the distribution of SNP density on the chromosomes.

**Fig. 7:**
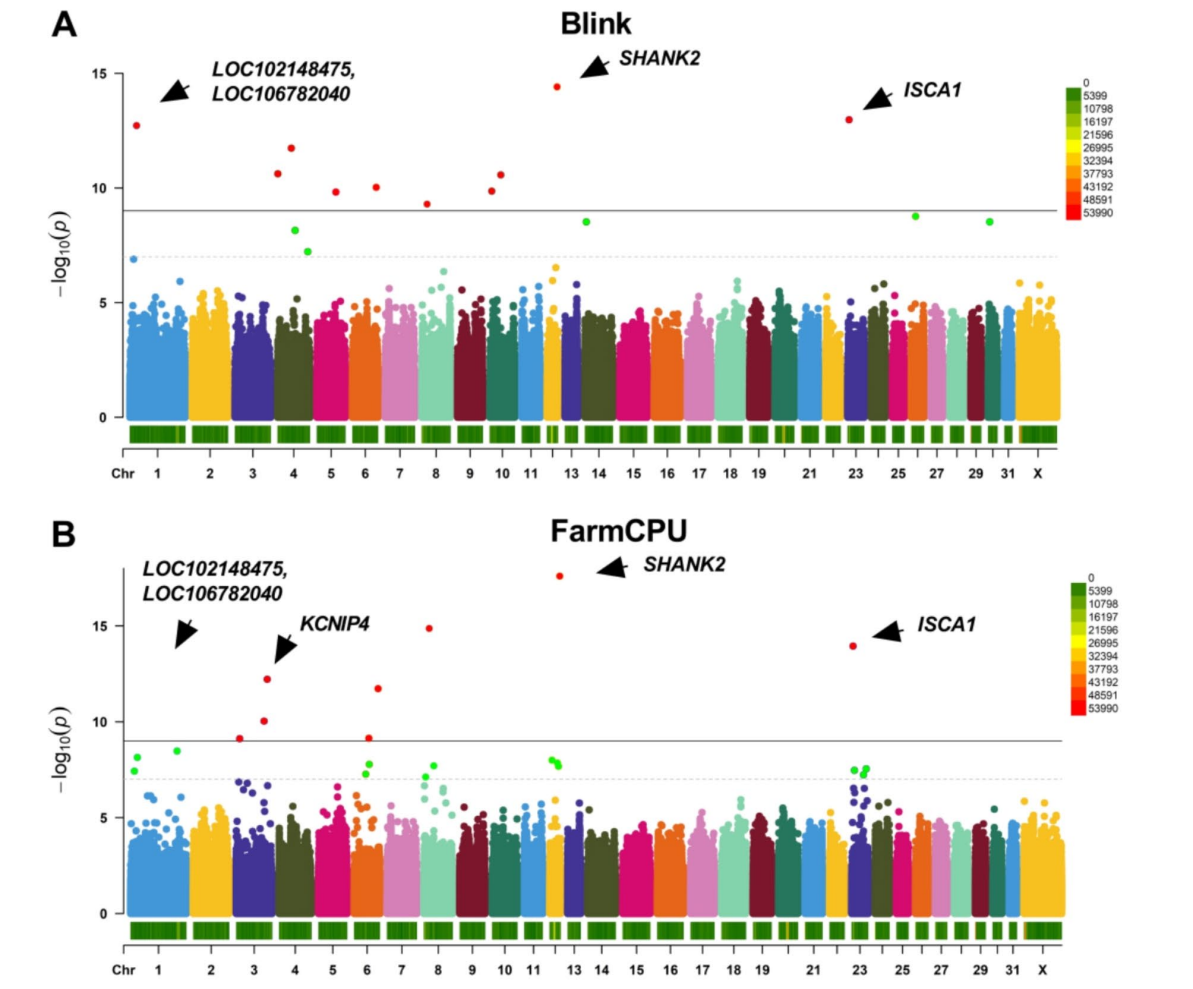
Manhattan plots of ranking score trait base on Blink (**A**) and FarmCPU (**B**). The values in the upper right corner of the image indicate the distribution of SNP density on the chromosomes.

The association loci were screened by GWAS, *P* values were -log10 transformed, and Manhattan plots were drawn (Figs. 6 and 7), with a total of 24 significant loci (*P* < 6.05 × 10^− 9^) and 22 suggested SNP markers (*P* < 1.21 × 10^− 7^). In the Blink model (Figs. 6A and 7A), eight SNP loci were found to be associated with the speed trait (*P* < 1.21 × 10^− 7^), of which five were significantly associated (*P* < 6.05 × 10^− 9^), and 18 SNP loci were found to be associated with the ranking score trait (*P* < 1.21 × 10^− 7^), with 13 SNP loci being significantly correlated (*P* < 6.05 × 10^− 9^). In the FarmCPU model (Figs. 6B and 7B), four SNP loci were found to be associated with the speed trait (*P* < 1.21 × 10^− 7^), with three of them significantly associated (*P* < 6.05 × 10^− 9^); 22 SNP loci were found to be associated with the ranking score trait (*P* < 1.21 × 10^− 7^), of which 10 SNP loci were significantly correlated (*P* < 6.05 × 10^− 9^).

### *P* value expansion detection

**Fig. 8:**
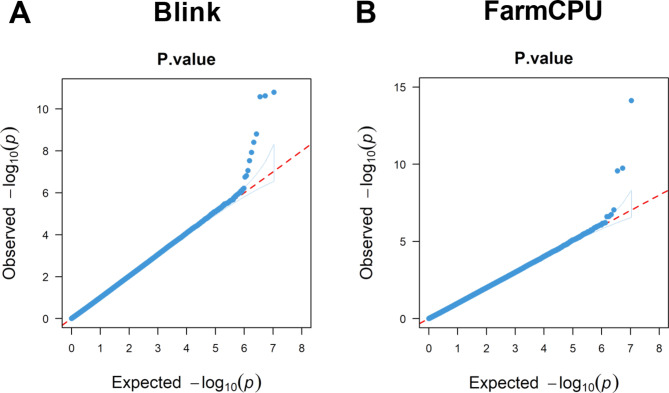
QQ plots of speed trait base on Blink (**A**) and FarmCPU (**B**).

**Fig. 9:**
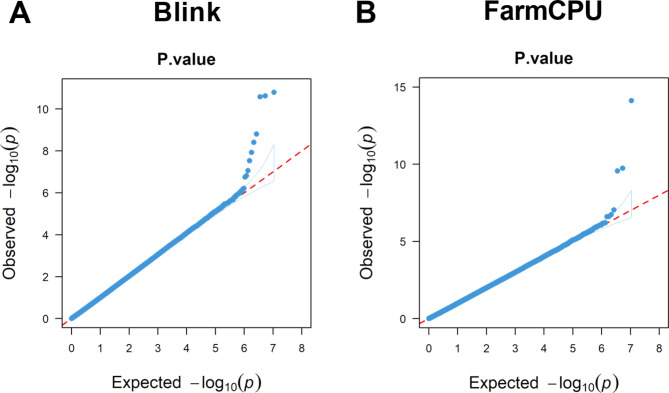
QQ plots of ranking score trait base on Blink (**A**) and FarmCPU (**B**).

To test for population inflation in the results of this GWAS, we compared the observed *P* values with the randomized expected *P* values (Figs. 8 and 9). The results show that most SNPs are on the diagonal (red symbols), which indicates that the population structure of this GWAS calculation was well controlled. The upward movement of significant loci was observed in both Blink and FarmCPU models, and the combined Q-Q results of the three models proved that the upward movement of loci was not caused by inflated *P* values and confirmed the validity of the results.

### Significant site information

**Fig. 10:**
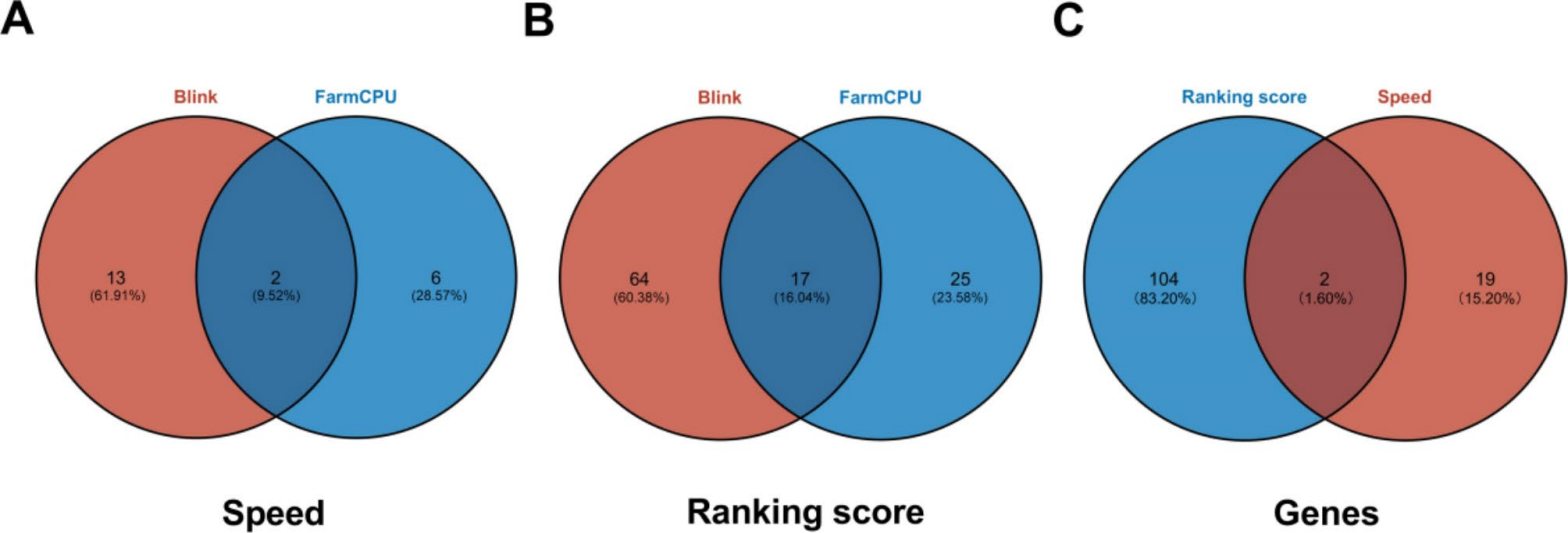
Venn Diagram of significant loci (**A**, **B**) and genes (**C**) for speed and ranking score traits.

Gene annotation was performed based on the screened SNP loci. Yili horses showed 125 functional genes for the racing performance traits of speed and ranking score, of which 48 were significant (*p* < 6.05 × 10^− 9^) (Tables 2 and 3; Fig. 10); detailed gene information is provided in the Supplementary Material( Tables S12-S13). There were two intersecting genes associated with the speed and 17 intersecting genes associated with the ranking score. The most significant loci were all related to speed and ranking score traits (ECA1: 22698579), with annotated genes *LOC102148475* and *LOC106782040*, and the proportion of variance explained (PVE) equal to 5.789.

**Table 2:** Data on significant loci for speed trait.

Chr	Position	Models	*P* value	PVE(%)	Gene
1	22,698,579	Blink, FarmCPU	1.61 × 10^− 11^, 7.60 × 10^− 15^	5.832	*LOC102148475*,* LOC106782040*
1	38,288,672	FarmCPU	1.84 × 10^− 10^	3.014	*LOC111770784*
1	114,866,932	FarmCPU	2.76 × 10^− 10^	1.103	*CYFIP1*,* HERC2*,* NIPA1*,* NIPA2*
8	74,314,792	Blink	1.58 × 10^− 9^	2.964	*DCC*
16	15,545,322	Blink	2.65 × 10^− 11^	2.278	*LOC100630775*,* CNTN6*
18	4,548,256	Blink	3.93 × 10^− 9^	7.112	*LOC100053131*
25	17,895,550	Blink	2.38 × 10^− 11^	4.074	*LOC100053653*,* SLC46A2*,* SNX30*

**Table 3:** Data on significant loci for ranking score trait.

Chr	Position	Models	*P* value	PVE(%)	Gene
1	22,698,579	Blink, FarmCPU	1.91 × 10^− 13^, 7.15 × 10^− 9^	5.832	*LOC102148475*,* LOC106782040*
3	94,454,562	FarmCPU	9.29 × 10^− 11^	0.549	*LOC111773008*
3	104,971,599	FarmCPU	6.03 × 10^− 13^	0.252	*KCNIP4*
3	11,432,659	FarmCPU	7.61 × 10^− 10^	1.147	*GOT2*,* SLC38A7*
4	250,019	Blink	2.41 × 10^− 11^	1.350	*LOC100053609*
4	46,271,300	Blink	1.85 × 10^− 12^	0.296	*LOC111773180*,* ETV1*
5	64,306,103	Blink	1.51 × 10^− 10^	0.366	*SNX7*,* LOC106783210*
8	17,931,378	Blink, FarmCPU	5.05 × 10^− 10^, 1.34 × 10^− 15^	0.894	*LOC111774796*,* LOC111774797*,* PEBP1*,* RFC5*,* TAOK3*,* VSIG10*,* WSB2*
10	6,205,418	Blink	1.37 × 10^− 10^	2.604	*ZNF30*,* ZNF792*
10	37,217,201	Blink	2.68 × 10^− 11^	1.399	*LOC100069941*,* LOC111775302*
12	33,662,348	Blink, FarmCPU	3.88 × 10^− 15^, 2.51 × 10^− 18^	0.628	*LOC111776027*,* SHANK2*
14	4,616,908	Blink	3.03 × 10^− 9^	0.494	*HK3*,* UNC5A*
23	4,362,189	Blink, FarmCPU	1.07 × 10^− 13^, 1.13 × 10^− 14^	0.199	*C23H9orf153*,* ISCA1*,* LOC106782456*,* ZCCHC6*
26	14,850,165	Blink	1.71 × 10^− 9^	0.639	*LOC102147870*,* LOC106782629*
30	4,951,289	Blink	3.00 × 10^− 9^	0.109	*KIF26B*

### Gene function enrichment analysis

**Fig. 11:**
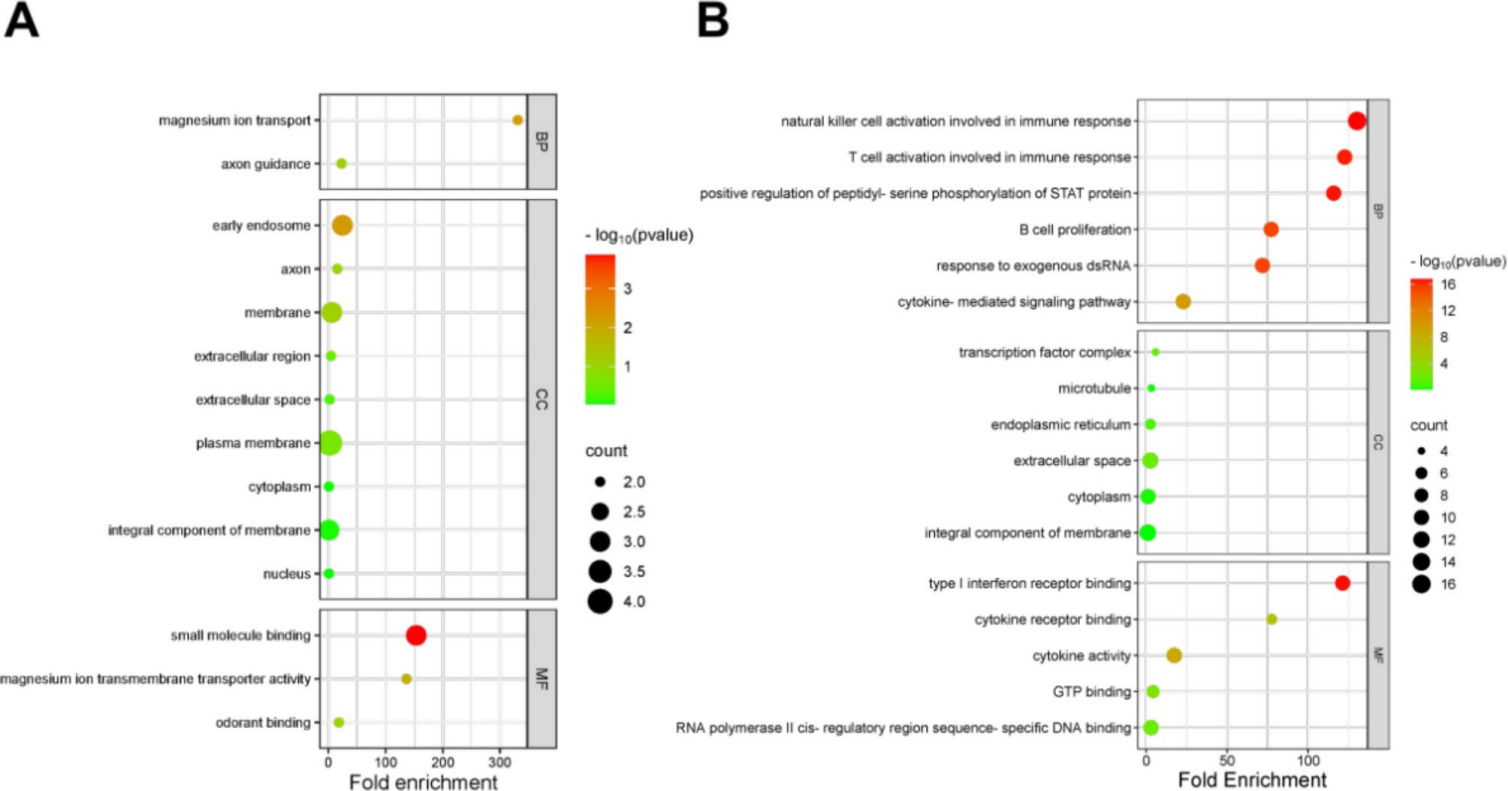
Enrichment determination of speed (**A**) and ranking score (**B**) traits associated genes by GO analysis.

**Fig. 12:**
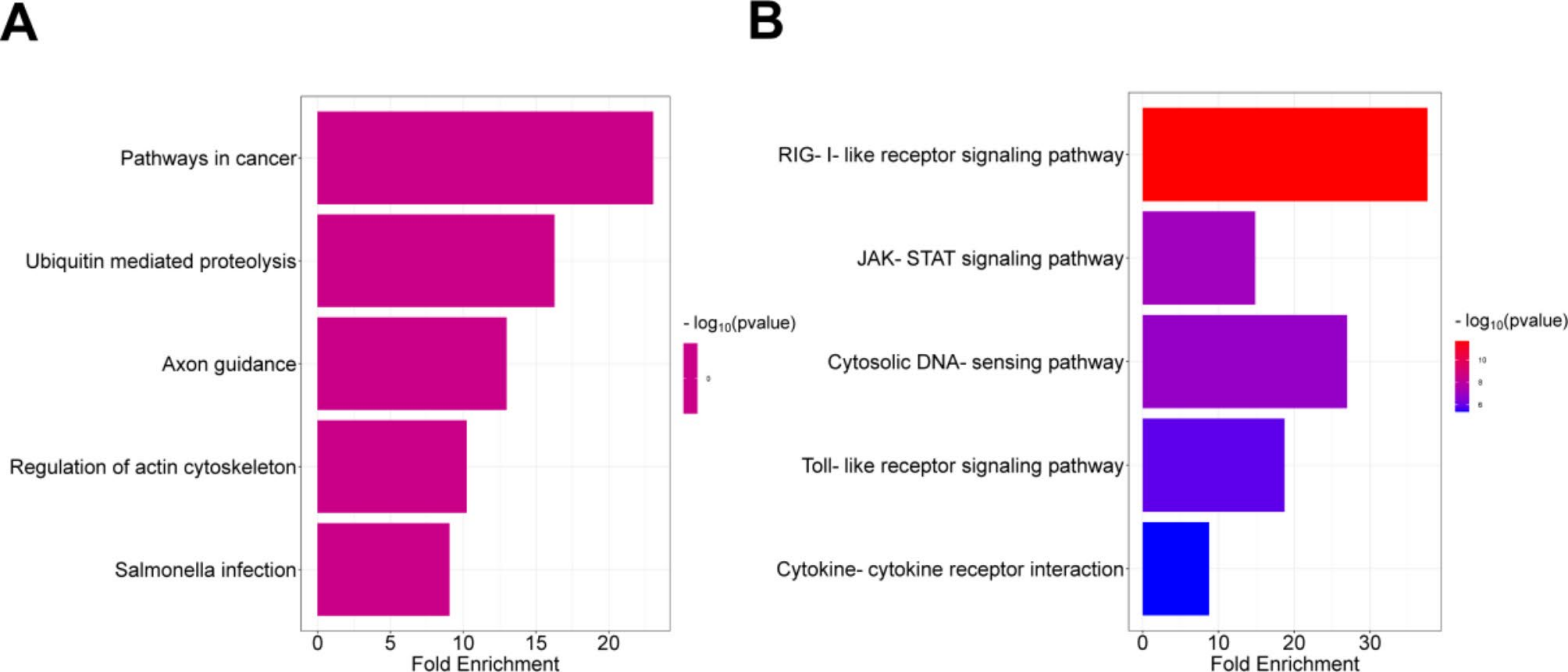
Enrichment determination of speed (**A**) and ranking score (**B**) traits associated genes by KEGG analysis.

The results of GO enrichment of genes related to speed trait showed (Fig. 11) that they were significantly enriched in magnesium ion transport, magnesium ion transmembrane transporter activity, and axon guidance. The KEGG results showed significant enrichment mainly in axon guidance and regulation of the actin cytoskeleton. The results of GO enrichment of genes related to ranking score trait showed (Fig. 12) significant enrichment mainly in immune regulation (natural killer cell activation in the immune response and T cell activation in the humoral immune response), cytokine receptor binding (type I interferon receptor binding, cytokine receptor binding). KEGG results showed significant enrichment mainly in the RIG-I-like receptor signaling pathway, the JAK-STAT signaling pathway, and cytokine-cytokine receptor interaction.

## Discussion

As an integrated tool for genomic association and prediction, GAPIT is being widely used in genome research due to its varied analytical strategies and functions^48–50^. In GAPIT (ver 3.0), FarmCPU and BLINK were evaluated and found to have extraordinary computational speed and statistical power^38^. In a comparison of several GWAS models, Jiabo Wang et al. used a comparison function to evaluate the computational power, FDR, and type I error of GLM, MLM, and FarmCPU models, and the results showed that FarmCPU outperformed MLM and GLM^36^. The BLINK, FarmCPU model used in this study had high sensitivity and statistical functionality, locating 46 genomic regions associated with racing performance traits in the Yili horse. These helped to identify mutated loci for higher racing performance and provided new insights on methods for detecting and selecting desirable genetic variations.

The locus most significantly associated with both speed and ranking score traits in Yili horses was ECA1: 22,698,579 (BLINK, FarmCPU, 1.61 × 10^− 11^, 7.60 × 10^− 15^; 1.91 × 10^− 13^, 7.15 × 10^− 9^), which is linked to the annotated genes *LOC102148475* and *LOC106782040*, whose functional roles are poorly understood or currently unknown. However, this SNP locus is closer to the only known functional locus for the speed trait (ECA1: 25885857) reported in the horse QTLdb database^51^. It is likely to be closely related to the racing performance of Yili horses and is thus valuable for further in-depth study.

The most significant locus for the speed trait was ECA16: 15,545,322 (BLINK, 2.65 × 10^− 11^), an SNP locus 100 kb away from the significant locus reported in the horse QTLdb database for the racing performance trait (ECA16: 15645555). This locus is located 190 kb downstream of the *CNTN6* gene, which encodes a glycosylphosphatidylinositol (GPI)-anchored neuronal membrane protein that is a member of the immunoglobulin superfamily and may play a role in the formation of axonal connections in the developing nervous system^52^. Studies have shown that a deficiency of *CNTN6* in mice leads to severe motor coordination abnormalities and learning difficulties^53^. Motor coordination is crucial for high-speed performance in Yili horses, especially during a race, where the ability to coordinate between limbs is essential for the fastest speed.

The second significant site is ECA1: 114,866,932 (FarmCPU, 2.76 × 10^− 10^). This locus is located 15 kb upstream of *NIPA1*. The *NIPA1* gene encodes a magnesium transporter, which is associated with early nuclear endosomes and cell surfaces in various types of neurons and in epithelial cells. The protein may play a role in the development and maintenance of the nervous system. It has been shown that mutations in this gene are associated with degenerative motor neuron diseases^54^. Therefore, the *NIPA1* gene may be closely related to motor neuron development and control during high-intensity activity in Yili horses.

The third significant site is ECA8: 74314792 (BLINK, 1.58 × 10^− 9^), which is located within the *DCC* gene, near the 5’ end. The product of *DCC* gene expression is a transmembrane phosphoprotein, which is a member of the immunoglobulin superfamily of cell adhesion molecules. The amino acid sequence of *DCC* shares homology with neural cell adhesion factor (NCAM) and other related cell surface glycoproteins, which suggests that loss of *DCC* function may lead to decreased cell-to-cell contact and adhesion, thus enhancing the metastatic ability of cancer cells^55^. It has been shown that *DCC* can encode netrin 1 receptors and mediate axon guidance of neuronal growth cones towards the source of netrin 1 ligands^56^, a process that has been linked to the development of adolescent dopamine neurons^57^. Horse racing is a high-intensity sport with critical neuronal involvement, and *DCC* may be involved in neuronal development in racehorses by regulating the excitatory conduction mechanism, which in turn could affect racing performance.

The most significant locus in the ranking score trait is ECA12: 33662348 (BLINK, FarmCPU, 3.88 × 10^− 15^, 2.51 × 10^− 18^), which is 26 kb upstream of *SHANK2*, near the 5’ end. The *SHANK2* gene enables ionotropic glutamate receptor binding activity, which is involved in the regulation of chemical synaptic transmission and synaptic organization of multiple processes, including learning and memory. Located in a variety of cellular components and expressed in the cerebral cortex, *SHANK2* encodes a scaffolding protein in the postsynaptic membrane of excitatory neurons and is involved in the induction and maturation of dendritic spines^58^. Competitive racing performance is also an ability acquired with constant practice, and the *SHANK2* gene may be associated with competitive neurotransmission and reinforcement processes, the lack of which could result in loss of competitive racing performance in racehorses.

The second significant site is ECA23: 4362189 (BLINK, FarmCPU, 1.07 × 10^− 13^, 1.13 × 10^− 14^), which is 33 kb upstream of the *ISCA1* gene near the 5’ end. The *ISCA1* gene codes for a mitochondrial protein involved in the biogenesis and assembly of iron-sulfur clusters, which play a role in electron transfer. Studies have shown that *ISCA1* gene deletion leads to abnormal morphology and impaired enzyme activity of mitochondrial respiratory chain complexes I, II and IV, and reduced ATP synthesis, concurrent with signs of dilated cardiomyopathy^59^. In horse racing, the strength of cardiac function tends to determine the magnitude of the ability to exercise–a strong heart is a prerequisite for high-intensity exercise. Thus, the *ISCA1* gene may influence heart function by affecting mitochondrial proteins, enzyme activities, and ATP synthesis, which in turn indirectly affects horse racing performance.

The third significant site is ECA3: 104971599 (FarmCPU, 6.03 × 10^− 13^), near the 5’ end within the *KCNIP4* gene, which is a member of the family of potassium-ion (Kv) channel-interacting proteins (*KCNIPs*), which share similarities with the calcium-binding proteins. It regulates neuronal excitability in response to changes in intracellular calcium ions by modulating A-type currents and thus neuronal excitability^60^. Related studies have reported that the *KCNIP4* gene is associated with growth traits in broiler chickens^61^, sheep^62^, and beef cattle^63^. Potassium ion channels are involved in the regulation of a variety of neuronal functions. Strenuous exercise is accompanied by a complex physiological regulatory process. During intense exercise of short duration, the body enhances the loading capacity of exercise vectors through neurotransmitter release, accelerated heart rate, insulin secretion, and modulation of neuronal excitability. *KCNIP4* may be involved in this process by improving the efficiency and capacity of neural activity, thus allowing a rapid burst of physical exertion.

In addition, the ranking score trait is associated with genes such as *GOT2* (glutamic acid transaminase) and *SLC38A7* (amino acid transporter protein), which are involved in the metabolism of amino acids^64^, which may provide an energy source for intense exertion.

This study has some limitations, such as the small sample size and the lack of information about SNP loci. The functions of the significant genes, *LOC102148475*, *LOC106782040*, *LOC111774796*, and *LOC111774797*, have not yet been identified, and further studies are needed to investigate the significant SNP loci.

## Conclusions

In this study, two GWAS models, BLINK, and FarmCPU, were used to analyze the racing performance traits of Yili horses, and a total of 46 SNP markers (24 significant markers) were associated with the BLINK and FarmCPU models, including 50 significant candidate genes. The discovery of some associated candidate genes (*CNTN6*,* NIPA1*,* DCC*,* SHANK2*,* ISCA1*,* KCNIP4*) will help us to understand the genetic mechanism. In addition, this study identified a locus (ECA1, 22698579) that is significantly associated with the traits of speed and ranking score in Yili horses. However, the specific function of this locus is not well understood and needs to be further explored. In conclusion, further research is needed to validate and expand upon the associations revealed in this study, as well as to explore the potential of using these genes to improve the genetics of racing performance in Yili horses.

## Electronic supplementary material

Below is the link to the electronic supplementary material.

Supplementary Material 1.

## Data Availability

The data that support the findings of this study are available from the corresponding author, C.W., upon reasonable request.
