# Supplementary material for: A genome-wide association study of the racing performance traits in Yili horses based on Blink and FarmCPU models
Source: Sci Rep. 2024 Nov 12;14:27648. doi: 10.1038/s41598-024-79014-w (PMC11557848; doi:10.1038/s41598-024-79014-w)
Supplement: Supplementary file 1 — Supplementary Material 1. [file 41598_2024_79014_MOESM1_ESM.docx]

**Supplementary Materials**

**Figure S1.** Genotype analysis including frequency of marker.

**
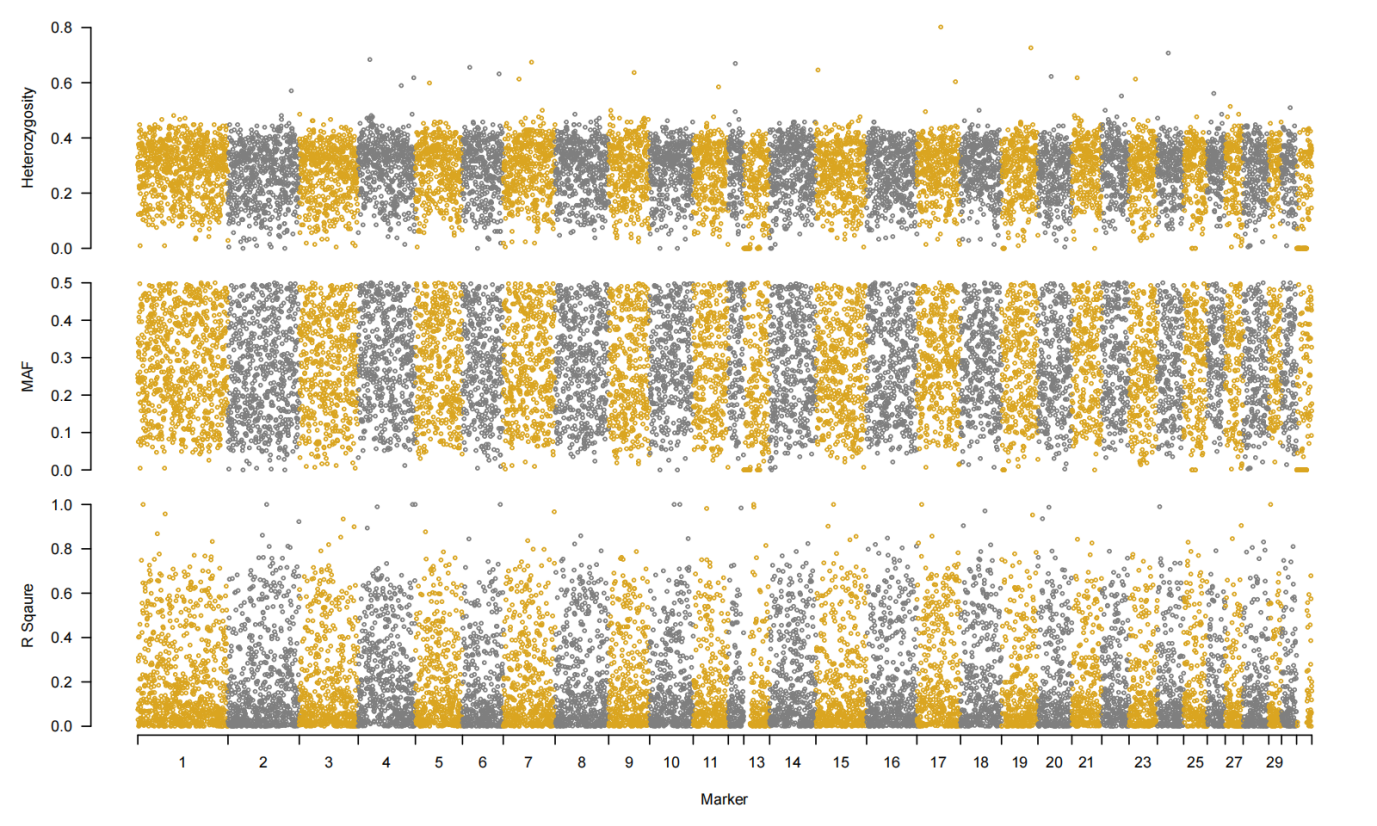
**

**Figure S2.** Genotype analysis including heterozygosity (a and b) and MAF (c).

**
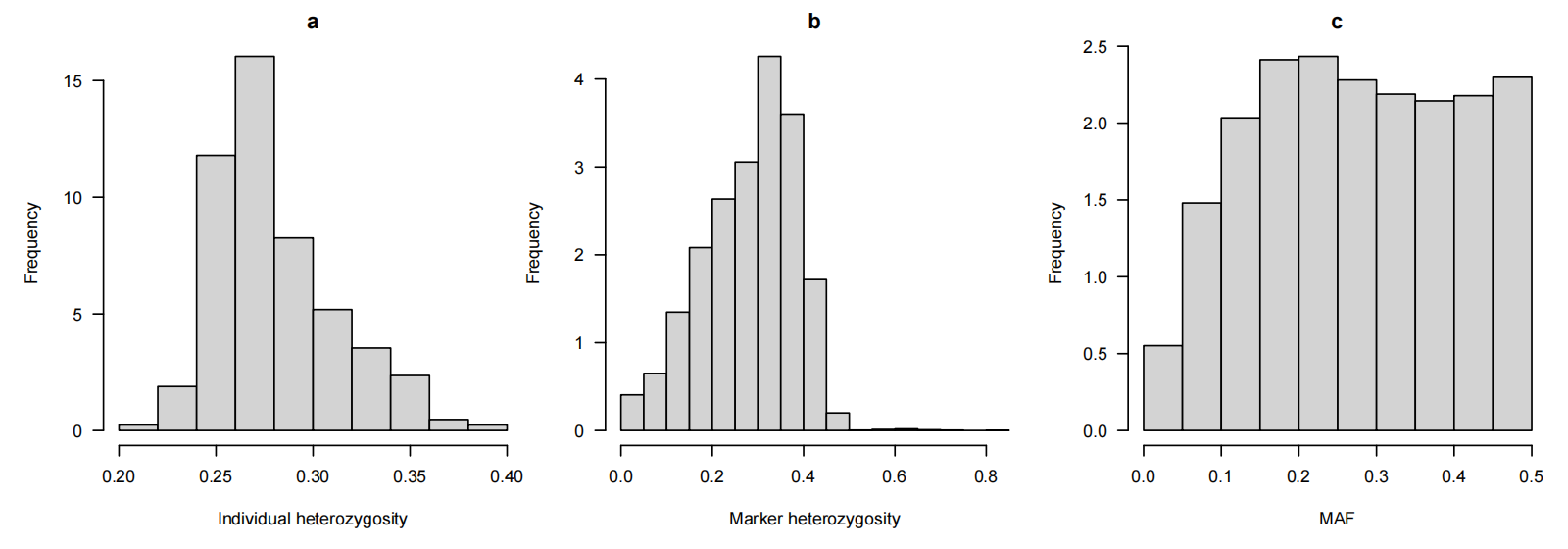
**

**Figure S3.** Principal component analysis (PC1 *vs.* PC2) of sample groups (TB, thoroughbred; YH, Yili horses).

**
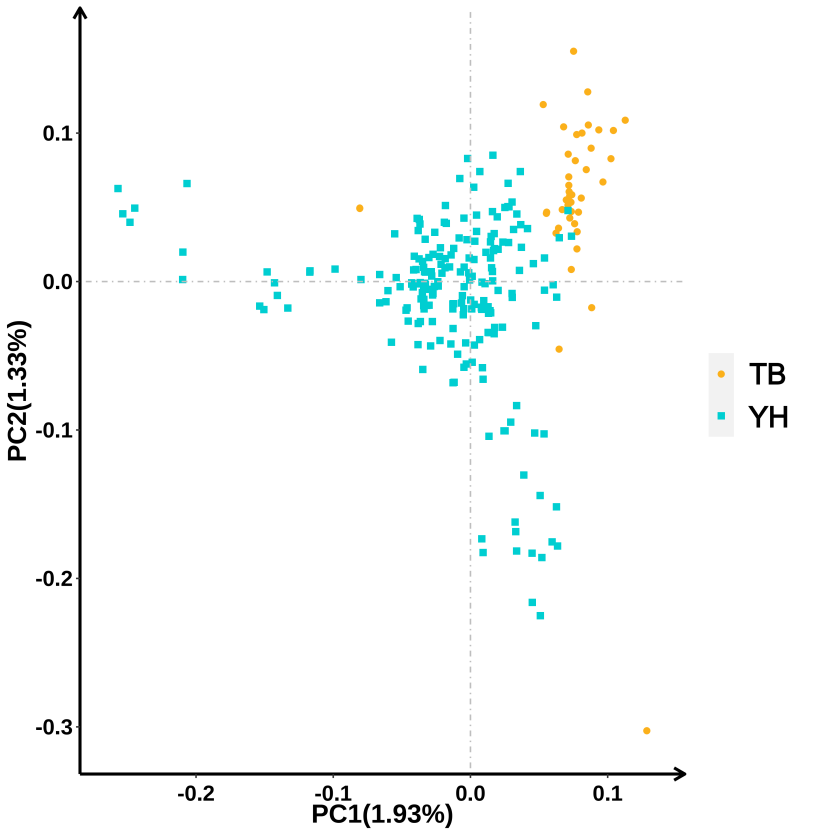
**

**Figure S4.** Principal component analysis (PC1 *vs.* PC3) of sample groups (TB, thoroughbred; YH, Yili horses).

**
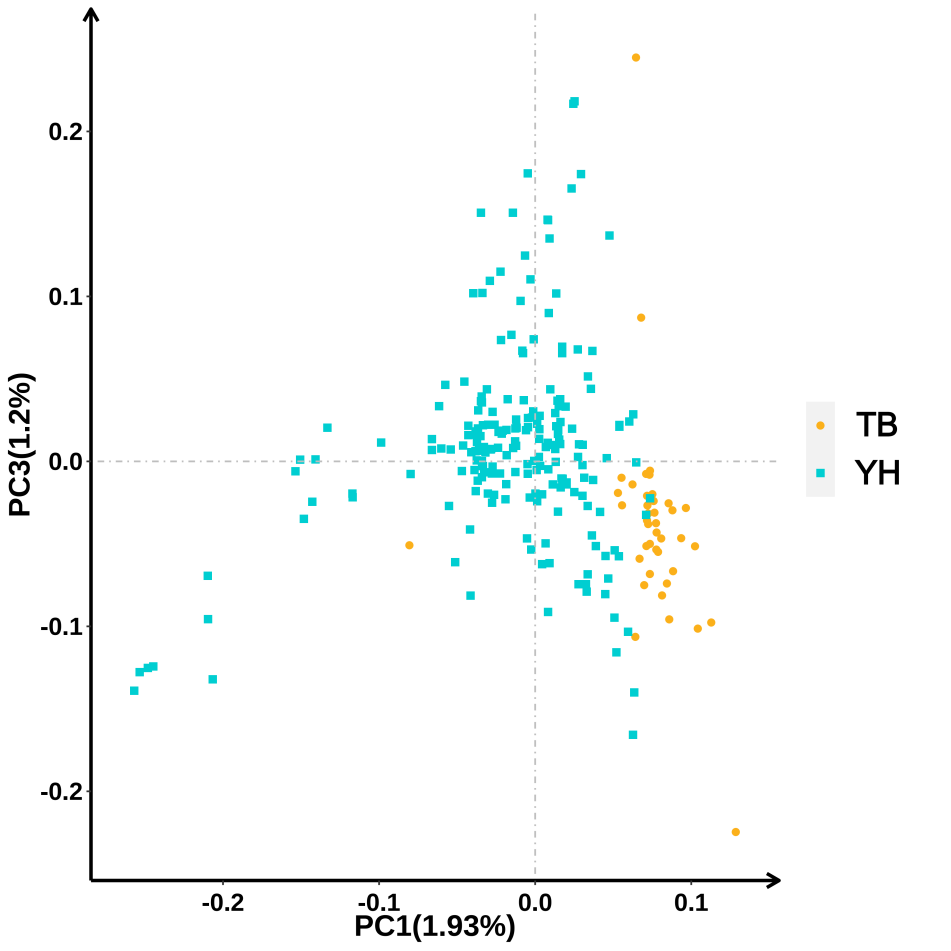
**

**Figure S5.** Principal component analysis (PC2 *vs.* PC3) of sample groups (TB, thoroughbred; YH, Yili horses).

**
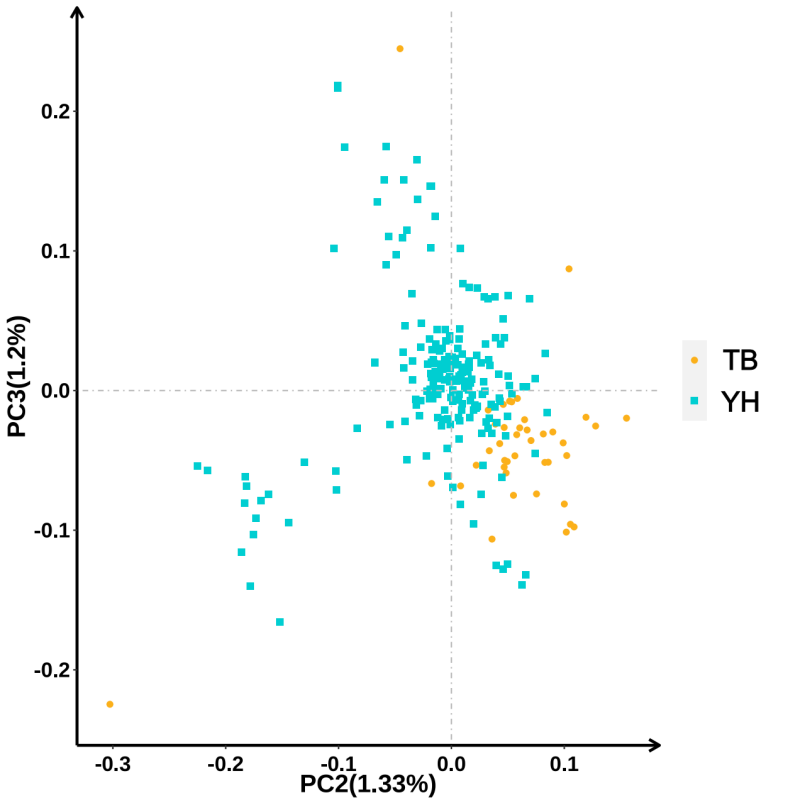
**

**Table S1.** Descriptive statistics of racing performance traits in Yili horses.

| **Traits** | **Number** | **Unit** | **Average** | **SD** | **Min** | **Max** | **CV(%)** |
| --- | --- | --- | --- | --- | --- | --- | --- |
| AS | 2576 | meter/second | 12.201 | 1.092 | 7.961 | 14.908 | 11.176 |
| RS | 2576 | score | 78.144 | 19.375 | 5.460 | 110.000 | 4.033 |

AS = average speed, RS = ranking score.

**Table S2.** Genetic correlation and phenotypic of racing performance of Yili horse. Standard errors of traits are reported in an extended version of the table.

| **Traits** | **AS** | **RS** |
| --- | --- | --- |
| **AS** | 1 | 0.735(0.009) |
| **RS** | 0.920(0.014) | 1 |

AS = average speed; RS = ranking score.

**Table S3.** Effect of different factors on racing performance traits.

| **Traits** | **Age^1)^** | | **Distance** | | **Year** | | **Gender** | | **Month** | | **Race class** | |
| --- | --- | --- | --- | --- | --- | --- | --- | --- | --- | --- | --- | --- |
|  | **df** | **F^2)^** | **df** | **F** | **df** | **F** | **df** | **F** | **df** | **F** | **df** | **F** |
| AS | 3 | 33.62^**^ | 5 | 163.90^**^ | 11 | 13.59^**^ | 1 | 12.55^**^ | 9 | 6.96^**^ | 2 | 13.72^**^ |
| RS | 3 | 1.38^*^ | 5 | 3.54^**^ | 11 | 2.27^**^ | 1 | 10.62^**^ | 9 | 1.03 | 2 | 3.00^*^ |

AS = average speed; RS = ranking score.

^1)^ Age: age of racing; Distance: distance of racing; Year: year of birth; Gender: gender of horse; Month: month of racing; Race class: level of racing.

^2)^ Effect of age, distance, year, gender, month, and race class on racing performance traits.

* *p*<0.05, ** *p*<0.01.

**Table S4.** Effect of horse age on racing performance traits.

| **Age** | **Number** | **AS (m/s)^1)^** | **RS (score)** |
| --- | --- | --- | --- |
| 2 | 1066 | 11.68±0.08^2)c^ | 81.76±2.38^a^ |
| 3 | 680 | 11.69±0.06^c3)^ | 78.13±2.08^b^ |
| 4 | 442 | 12.04±0.07^b^ | 79.55±2.19^ab^ |
| 5 | 388 | 12.21±0.07^a^ | 79.31±2.20^ab^ |

AS = average speed; RS = ranking score.

^1)^ Units of traits are shown in parentheses.

^2)^ Phenotypic data of different herds are displayed as mean ± standard deviation.

^3)^ Different superscript letters (a, b) in the same column mean significant differences (*P*<0.05).

**Table S5.** Effect of distance on racing performance traits.

| **Distance** | **Number** | **AS (m/s)^1)^** | **RS (score)** |
| --- | --- | --- | --- |
| 1000 | 687 | 13.04±0.07^2)a^ | 83.55±2.22^a^ |
| 1600 | 434 | 12.67±0.07^b3)^ | 80.10±2.22^a^ |
| 2000 | 509 | 11.68±0.07^c^ | 80.80±2.24^a^ |
| 3000 | 181 | 11.50±0.08^d^ | 79.33±2.41^ab^ |
| 3600 | 348 | 11.20±0.08^e^ | 74.61±2.45^b^ |
| 5000 | 417 | 11.35±0.08^d^ | 79.74±2.57^a^ |

AS: average speed; RS: ranking score.

^1)^ The unit of traits are shown in parentheses.

^2)^ Phenotypic data of different herds are displayed as mean ± standard deviation.

^3)^ Different superscript letters (a, b) in the same column mean significant differences (*P*<0.05).

**Table S6.** Effect of year on racing performance traits.

| **Year** | **Number** | **AS (m/s)^1)^** | **RS (score)** |
| --- | --- | --- | --- |
| 2010 | 10 | 11.13±0.28^2)e^ | 73.68±7.61^a^ |
| 2011 | 12 | 12.12±0.30^abc3)^ | 94.78±9.38^a^ |
| 2012 | 35 | 11.57±0.16^c^ | 85.41±4.58^a^ |
| 2013 | 241 | 11.66±0.07^de^ | 79.71±2.22^abc^ |
| 2014 | 360 | 11.81±0.06^c^ | 81.87±2.00^a^ |
| 2015 | 306 | 11.95±0.07^b^ | 79.32±2.18^abc^ |
| 2016 | 680 | 11.94±0.06^b^ | 79.84±1.85^ab^ |
| 2017 | 333 | 12.33±0.06^a^ | 81.54±1.99^a^ |
| 2018 | 296 | 12.21±0.07^a^ | 75.63±2.07^cd^ |
| 2019 | 137 | 11.73±0.08^cd^ | 74.07±2.51^d^ |
| 2020 | 65 | 12.11±0.11^ab^ | 73.97±3.20^bcd^ |
| 2021 | 101 | 12.31±0.10^a^ | 76.46±2.97^abcd^ |

AS = average speed; RS = ranking score.

^1)^ The units of traits are shown in parentheses.

^2)^ Phenotypic data of different herds are displayed as mean ± standard deviation.

^3)^ Different superscript letters (a, b) in the same column mean significant differences (*P*<0.05).

**Table S7.** Effect of gender on racing performance traits.

| **Gender** | **Number** | **AS (m/s)^1)^** | **RS (score)** |
| --- | --- | --- | --- |
| 1 | 1827 | 12.03±0.08^2)b^ | 77.81±1.79^b^ |
| 2 | 749 | 12.15±0.08^a3)^ | 81.57±2.01^a^ |

AS = average speed; RS: ranking score.

^1)^ The units of traits are shown in parentheses.

^2)^ Phenotypic data of different herds are displayed as mean ± standard deviation.

^3)^ Different superscript letters (a, b) in the same column mean significant differences (*P*<0.05).

**Table S8.** Effect of months on racing performance traits.

| **Months** | **Number** | **AS (m/s)^1)^** | **RS (score)** |
| --- | --- | --- | --- |
| 3 | 7 | 10.41±0.31^2)d^ | 80.41±9.15^ab^ |
| 4 | 50 | 11.97±0.13^bc3)^ | 74.92±4.44^ab^ |
| 5 | 75 | 12.04±0.11^bc^ | 80.15±2.19^ab^ |
| 6 | 322 | 11.85±0.07^c^ | 78.99±2.30^b^ |
| 7 | 378 | 12.03±0.07^b^ | 79.58±1.98^ab^ |
| 8 | 648 | 12.06±0.06^b^ | 84.05±2.40^a^ |
| 9 | 331 | 12.05±0.07^b^ | 80.64±2.44^ab^ |
| 10 | 285 | 12.04±0.07^b^ | 80.73±2.13^ab^ |
| 11 | 319 | 12.30±0.07^a^ | 81.27±4.41^ab^ |
| 12 | 102 | 11.99±0.09^bc^ | 76.15±4.22^ab^ |

AS = average speed; RS = ranking score.

^1)^ The units of traits are shown in parentheses.

^2)^ Phenotypic data of different herds are displayed as mean ± standard deviation.

^3)^ Different superscript letters (a, b) in the same column mean significant differences (*P*<0.05).

**Table S9.** Effect of race class on racing performance traits.

| **Race class** | **Number** | **AS (m/s)^1)^** | **RS (score)** |
| --- | --- | --- | --- |
| 1 | 1408 | 11.74±0.06^2)b^ | 76.90±1.81^b^ |
| 2 | 523 | 11.94±0.07^a3)^ | 81.25±2.15^a^ |
| 3 | 645 | 12.04±0.07^a^ | 80.92±2.33^a^ |

AS: average speed; RS: ranking score.

^1)^ The units of traits are shown in parentheses.

^2)^ Phenotypic data of different herds are displayed as mean ± standard deviation.

^3)^ Different superscript letters (a, b) in the same column mean significant differences (*P*<0.05).

**Table S10.** Statistics of sequencing data evaluation.

| **Sample** | **Raw Reads Number** | **Clean Reads Number** | **Raw Bases**  **(bp)** | **Clean Bases(bp)** | **Effective Rate(%)** | **Q20(%)** | **Q30(%)** |
| --- | --- | --- | --- | --- | --- | --- | --- |
| 22Z001 | 237699744 | 237649484 | 35654961600 | 35282752798 | 98.96 | 96.14 | 88.73 |
| 22Z005 | 252370112 | 252328184 | 37855516800 | 37484288482 | 99.02 | 98.19 | 93.63 |
| 22Z008 | 307014564 | 306945510 | 46052184600 | 45384231750 | 98.55 | 97.81 | 92.95 |
| 22Z009 | 209914802 | 209877868 | 31487220300 | 31169509360 | 98.99 | 97.07 | 91.09 |
| 22Z010 | 263424654 | 263341990 | 39513698100 | 39188150910 | 99.18 | 97.63 | 92.71 |
| 22Z018 | 235534796 | 235505152 | 35330219400 | 35044430264 | 99.19 | 96.93 | 90.75 |
| 22Z021 | 192114314 | 192090670 | 28817147100 | 28436826472 | 98.68 | 97.58 | 92.56 |
| 22Z022 | 268865700 | 268806094 | 40329855000 | 39906758184 | 98.95 | 97.28 | 91.62 |
| 22Z024 | 229930338 | 229894684 | 34489550700 | 34135517876 | 98.97 | 97.59 | 92.57 |
| 22Z025 | 270026742 | 269981464 | 40504011300 | 40107521790 | 99.02 | 97.96 | 93.36 |
| 22Z029 | 266626066 | 266527926 | 39993909900 | 39573756840 | 98.95 | 97.68 | 92.85 |
| 22Z031 | 262368810 | 262269028 | 39355321500 | 38989937434 | 99.07 | 98.14 | 94.19 |
| 22Z032 | 250217764 | 250193668 | 37532664600 | 37039605946 | 98.69 | 96.76 | 90.23 |
| 22Z040 | 199372432 | 199348390 | 29905864800 | 29570992766 | 98.88 | 97.29 | 91.16 |
| 22Z043 | 208973550 | 208923096 | 31346032500 | 31033740720 | 99.00 | 96.38 | 89.02 |
| 22Z047 | 227262982 | 227182710 | 34089447300 | 33760168612 | 99.03 | 97.85 | 93.15 |
| 22Z058 | 207641736 | 207595960 | 31146260400 | 30884541498 | 99.16 | 96.68 | 89.89 |
| 22Z061 | 227034836 | 226976206 | 34055225400 | 33785585332 | 99.21 | 98.02 | 93.76 |
| 22Z063 | 214057546 | 214023930 | 32108631900 | 31797678792 | 99.03 | 98.09 | 93.58 |
| 22Z085 | 225518630 | 225467164 | 33827794500 | 33455354518 | 98.90 | 96.47 | 88.87 |
| 22Z086 | 229295122 | 229269764 | 34394268300 | 34071057024 | 99.06 | 96.39 | 88.59 |
| 22Z036 | 227678342 | 227573560 | 34151751300 | 33777397486 | 98.90 | 98.05 | 93.55 |
| 22Z088 | 197700252 | 197645008 | 29655037800 | 29453621594 | 99.32 | 97.41 | 91.23 |
| 22Z091 | 257743048 | 257691272 | 38661457200 | 38394401620 | 99.31 | 97.21 | 90.50 |
| W1_1 | 201260634 | 201242380 | 30189095100 | 29779030418 | 98.64 | 96.72 | 90.00 |
| W1_2 | 103600742 | 103571238 | 15540111300 | 15447455286 | 99.40 | 97.77 | 93.31 |
| W1_3 | 146285042 | 146250848 | 21942756300 | 21627459878 | 98.56 | 94.28 | 83.88 |
| W1_4 | 95529752 | 95511058 | 14329462800 | 14245693234 | 99.42 | 97.31 | 91.99 |
| W1_5 | 96568750 | 96544798 | 14485312500 | 14387843494 | 99.33 | 96.92 | 90.67 |
| W1_6 | 152119162 | 152090102 | 22817874300 | 22533153602 | 98.75 | 97.38 | 91.65 |
| W1_7 | 119014980 | 118988730 | 17852247000 | 17661614278 | 98.93 | 96.67 | 89.86 |
| W1_8 | 139574616 | 139561720 | 20936192400 | 20708497234 | 98.91 | 97.32 | 91.61 |
| W1_9 | 113586696 | 113567532 | 17038004400 | 16847599682 | 98.88 | 95.69 | 87.33 |
| W1_10 | 132159876 | 132125222 | 19823981400 | 19568096000 | 98.71 | 96.26 | 88.77 |
| W1_11 | 138780208 | 138733122 | 20817031200 | 20477597956 | 98.37 | 97.14 | 91.19 |
| W1_12 | 117195636 | 117172462 | 17579345400 | 17432727862 | 99.17 | 97.64 | 92.35 |
| W1_13 | 186152758 | 186105450 | 27922913700 | 27579497842 | 98.77 | 98.05 | 93.63 |
| W1_14 | 147105554 | 147093108 | 22065833100 | 21843253876 | 98.99 | 96.68 | 89.88 |
| W1_16 | 97887788 | 97859514 | 14683168200 | 14591221862 | 99.37 | 97.08 | 91.37 |
| W1_17 | 99167338 | 99134932 | 14875100700 | 14789136982 | 99.42 | 96.96 | 90.90 |
| W1_18 | 191014374 | 190993688 | 28652156100 | 28208926706 | 98.45 | 97.31 | 91.68 |
| W1_19 | 171020364 | 170974346 | 25653054600 | 25257331550 | 98.46 | 95.73 | 87.44 |
| W1_20 | 161190166 | 161157546 | 24178524900 | 23864033772 | 98.70 | 96.93 | 90.46 |
| W1_21 | 136238492 | 136205026 | 20435773800 | 20118188136 | 98.45 | 97.35 | 91.74 |
| W1_22 | 134382850 | 134371566 | 20157427500 | 19932304212 | 98.88 | 95.83 | 87.69 |
| W1_23 | 108478546 | 108454682 | 16271781900 | 16026366356 | 98.49 | 96.52 | 89.45 |
| W1_24 | 141404054 | 141357198 | 21210608100 | 20931973884 | 98.69 | 97.14 | 91.03 |
| W1_25 | 140576290 | 140527690 | 21086443500 | 20850044912 | 98.88 | 97.70 | 92.67 |
| W1_26 | 135322334 | 135285456 | 20298350100 | 20032801416 | 98.69 | 97.64 | 92.44 |
| W1_27 | 156680518 | 156646240 | 23502077700 | 23178206730 | 98.62 | 96.07 | 88.28 |
| W1_28 | 148880486 | 148865630 | 22332072900 | 22012379892 | 98.57 | 97.00 | 90.79 |
| W1_29 | 96777040 | 96711384 | 14516556000 | 14401211038 | 99.21 | 97.66 | 92.55 |
| W1_30 | 111766352 | 111696000 | 16764952800 | 16643965830 | 99.28 | 97.55 | 92.42 |
| W1_32 | 98461500 | 98433226 | 14769225000 | 14667903006 | 99.31 | 97.48 | 92.33 |
| W1_33 | 104131952 | 104099092 | 15619792800 | 15495941618 | 99.21 | 97.17 | 91.61 |
| W1_34 | 100524700 | 100496048 | 15078705000 | 14982975034 | 99.37 | 96.67 | 90.13 |
| W1_35 | 100347460 | 100311636 | 15052119000 | 14950759172 | 99.33 | 97.45 | 92.07 |
| W1_36 | 120853016 | 120764350 | 18127952400 | 17960078068 | 99.07 | 97.59 | 92.55 |
| W1_37 | 118624730 | 118587264 | 17793709500 | 17661247602 | 99.26 | 97.09 | 91.43 |
| W1_38 | 97488822 | 97462930 | 14623323300 | 14531320728 | 99.37 | 97.33 | 91.79 |
| W1_39 | 100181062 | 100153376 | 15027159300 | 14925156836 | 99.32 | 97.38 | 92.07 |
| W1_40 | 96963146 | 96897540 | 14544471900 | 14437781872 | 99.27 | 97.59 | 92.50 |
| W1_41 | 106083824 | 106009022 | 15912573600 | 15803184250 | 99.31 | 97.44 | 92.10 |
| W1_42 | 105207370 | 105180800 | 15781105500 | 15667124946 | 99.28 | 97.23 | 91.76 |
| W1_43 | 97250822 | 97231198 | 14587623300 | 14494006110 | 99.36 | 97.24 | 91.55 |
| W1_45 | 95308048 | 95285884 | 14296207200 | 14184965928 | 99.22 | 97.14 | 91.54 |
| W1_46 | 100613506 | 100545730 | 15092025900 | 14977806474 | 99.24 | 97.55 | 92.33 |
| W1_47 | 98794216 | 98720710 | 14819132400 | 14709784822 | 99.26 | 97.66 | 92.71 |
| W1_48 | 101092162 | 101025674 | 15163824300 | 15063547862 | 99.34 | 96.84 | 90.38 |
| W1_49 | 108818322 | 108740476 | 16322748300 | 16196472172 | 99.23 | 97.79 | 93.15 |
| W1_50 | 102531226 | 102499716 | 15379683900 | 15275419910 | 99.32 | 97.33 | 92.05 |
| W1_51 | 114233558 | 114207104 | 17135033700 | 16992499922 | 99.17 | 97.43 | 92.37 |
| W1_52 | 99858362 | 99792848 | 14978754300 | 14867650324 | 99.26 | 97.44 | 92.00 |
| W1_54 | 99275876 | 99208968 | 14891381400 | 14772338294 | 99.20 | 97.71 | 92.79 |
| W1_55 | 100608138 | 100537548 | 15091220700 | 14980428120 | 99.27 | 97.44 | 92.04 |
| W1_56 | 97299846 | 97231652 | 14594976900 | 14510182370 | 99.42 | 97.12 | 91.13 |
| W1_57 | 100692830 | 100625596 | 15103924500 | 15015990924 | 99.42 | 96.98 | 90.77 |
| W1_58 | 106722734 | 106642720 | 16008410100 | 15877441816 | 99.18 | 97.80 | 93.17 |
| W1_59 | 100520494 | 100450102 | 15078074100 | 14962386106 | 99.23 | 97.77 | 93.00 |
| W1_60 | 106177958 | 106103686 | 15926693700 | 15800410310 | 99.21 | 97.25 | 91.60 |
| W1_61 | 107033480 | 106998128 | 16055022000 | 15930101360 | 99.22 | 97.59 | 92.77 |
| W1_62 | 100350914 | 100279700 | 15052637100 | 14943439498 | 99.27 | 97.67 | 92.72 |
| W1_63 | 109356472 | 109322552 | 16403470800 | 16279196088 | 99.24 | 97.04 | 91.27 |
| W1_64 | 114443810 | 114370888 | 17166571500 | 17032989052 | 99.22 | 97.49 | 92.21 |
| W1_65 | 100977166 | 100912352 | 15146574900 | 15048147514 | 99.35 | 97.52 | 92.29 |
| W1_66 | 118968038 | 118881346 | 17845205700 | 17671567096 | 99.03 | 97.86 | 93.35 |
| W1_67 | 98096652 | 98032502 | 14714497800 | 14589216806 | 99.15 | 97.39 | 91.97 |
| W1_68 | 99776614 | 99758958 | 14966492100 | 14854611444 | 99.25 | 97.23 | 91.58 |
| W1_69 | 119240296 | 119153032 | 17886044400 | 17758815886 | 99.29 | 97.88 | 93.42 |
| W1_70 | 108994228 | 108916330 | 16349134200 | 16222341840 | 99.22 | 97.39 | 92.02 |
| W1_71 | 110301656 | 110229380 | 16545248400 | 16409008090 | 99.18 | 97.97 | 93.65 |
| W1_72 | 100327728 | 100257998 | 15049159200 | 14944901292 | 99.31 | 97.95 | 93.53 |
| W1_75 | 95710074 | 95686946 | 14356511100 | 14251098878 | 99.27 | 97.55 | 92.64 |
| W1_76 | 141031446 | 140938914 | 21154716900 | 20905792856 | 98.82 | 98.05 | 93.90 |
| W1_78 | 96406780 | 96335794 | 14461017000 | 14349223232 | 99.23 | 97.60 | 92.57 |
| W1_79 | 98141038 | 98068674 | 14721155700 | 14612802560 | 99.26 | 97.57 | 92.48 |
| W1_80 | 102886082 | 102816748 | 15432912300 | 15315134878 | 99.24 | 96.98 | 90.85 |
| W1_81 | 98667438 | 98601680 | 14800115700 | 14681746494 | 99.20 | 97.75 | 92.88 |
| W1_83 | 105153568 | 105078080 | 15773035200 | 15633590938 | 99.12 | 97.30 | 91.74 |
| W1_84 | 100121292 | 100052234 | 15018193800 | 14922261464 | 99.36 | 97.65 | 92.65 |
| W1_85 | 125293828 | 125204274 | 18794074200 | 18642289620 | 99.19 | 97.37 | 91.92 |
| W1_86 | 99434214 | 99405150 | 14915132100 | 14797701872 | 99.21 | 97.23 | 91.56 |
| W1_87 | 103507782 | 103432984 | 15526167300 | 15423974368 | 99.34 | 97.61 | 92.60 |
| W1_88 | 96170402 | 96110182 | 14425560300 | 14323035422 | 99.29 | 97.59 | 92.50 |
| W1_89 | 100617848 | 100581734 | 15092677200 | 14981722576 | 99.26 | 97.36 | 92.06 |
| W1_90 | 95027752 | 95002476 | 14254162800 | 14131435098 | 99.14 | 96.59 | 88.84 |
| W1_91 | 100840734 | 100775148 | 15126110100 | 15033036138 | 99.38 | 97.23 | 91.40 |
| W1_92 | 117223314 | 117185394 | 17583497100 | 17451576738 | 99.25 | 97.36 | 92.15 |
| W1_93 | 100537734 | 100518570 | 15080660100 | 14965898820 | 99.24 | 97.11 | 91.36 |
| W1_94 | 96568418 | 96492616 | 14485262700 | 14378843370 | 99.27 | 97.52 | 92.33 |
| W1_95 | 99820920 | 99758126 | 14973138000 | 14889118602 | 99.44 | 96.77 | 90.13 |
| W1_96 | 101629876 | 101597770 | 15244481400 | 15125267644 | 99.22 | 96.98 | 91.11 |
| W1_97 | 99383944 | 99316874 | 14907591600 | 14812076154 | 99.36 | 97.75 | 92.85 |
| W1_98 | 117592160 | 117511764 | 17638824000 | 17515491196 | 99.30 | 97.36 | 91.88 |
| W1_99 | 98244232 | 98210374 | 14736634800 | 14641010238 | 99.35 | 97.29 | 91.67 |
| W1_100 | 98412552 | 98386720 | 14761882800 | 14658011800 | 99.30 | 96.61 | 89.81 |
| W1_101 | 100878408 | 100803988 | 15131761200 | 15023217702 | 99.28 | 97.46 | 92.16 |
| W1_102 | 95820486 | 95760560 | 14373072900 | 14272251382 | 99.30 | 97.56 | 92.38 |
| W1_103 | 100487560 | 100419182 | 15073134000 | 14961803774 | 99.26 | 97.71 | 92.85 |
| W1_104 | 108282162 | 108250260 | 16242324300 | 16120085866 | 99.25 | 97.18 | 91.66 |
| W1_105 | 114397948 | 114365844 | 17159692200 | 17023131004 | 99.20 | 97.46 | 92.43 |
| W1_106 | 95641232 | 95620288 | 14346184800 | 14240242530 | 99.26 | 97.14 | 91.51 |
| W1_107 | 99483466 | 99448412 | 14922519900 | 14802585782 | 99.20 | 97.45 | 91.99 |
| W1_108 | 105448182 | 105373890 | 15817227300 | 15710002804 | 99.32 | 97.69 | 92.78 |
| W1_109 | 123631330 | 123538614 | 18544699500 | 18404457556 | 99.24 | 97.51 | 92.38 |
| W1_110 | 100468112 | 100447006 | 15070216800 | 14950479112 | 99.21 | 97.31 | 91.84 |
| W1_112 | 97938154 | 97903680 | 14690723100 | 14573374440 | 99.20 | 97.23 | 91.80 |
| W1_113 | 99267182 | 99238272 | 14890077300 | 14773112348 | 99.21 | 97.38 | 92.09 |
| W1_114 | 102654244 | 102586054 | 15398136600 | 15267136548 | 99.15 | 97.55 | 92.41 |
| W1_115 | 103595484 | 103562078 | 15539322600 | 15422841272 | 99.25 | 96.97 | 91.07 |
| W1_116 | 119715286 | 119679138 | 17957292900 | 17820173028 | 99.24 | 97.10 | 91.45 |
| W1_117 | 100167066 | 100141364 | 15025059900 | 14908319908 | 99.22 | 97.22 | 91.57 |
| W1_118 | 134148054 | 134109002 | 20122208100 | 19954612972 | 99.17 | 97.29 | 91.96 |
| W1_119 | 112038680 | 112016410 | 16805802000 | 16706382818 | 99.41 | 97.21 | 91.69 |
| W1_120 | 104256192 | 104220336 | 15638428800 | 15515514388 | 99.21 | 97.14 | 91.52 |
| W1_121 | 103185430 | 103150670 | 15477814500 | 15383576090 | 99.39 | 96.85 | 90.72 |
| W1_122 | 97534118 | 97501476 | 14630117700 | 14533911652 | 99.34 | 97.42 | 92.32 |
| W1_123 | 97891158 | 97864720 | 14683673700 | 14589999996 | 99.36 | 97.13 | 91.25 |
| W1_124 | 120075284 | 120039436 | 18011292600 | 17860790314 | 99.16 | 97.49 | 92.59 |
| W1_125 | 102029140 | 101964670 | 15304371000 | 15202636252 | 99.34 | 97.45 | 92.09 |
| W1_126 | 101009460 | 100969434 | 15151419000 | 15048601696 | 99.32 | 97.13 | 91.41 |
| W1_127 | 114836156 | 114802718 | 17225423400 | 17108825348 | 99.32 | 97.70 | 93.12 |
| W1_128 | 100552982 | 100484490 | 15082947300 | 14984555492 | 99.35 | 97.52 | 92.24 |
| W1_129 | 100562864 | 100545150 | 15084429600 | 14990038356 | 99.37 | 97.15 | 91.47 |
| W1_130 | 99630278 | 99600180 | 14944541700 | 14846216494 | 99.34 | 97.30 | 91.80 |
| W1_131 | 100660884 | 100633436 | 15099132600 | 15008295664 | 99.40 | 97.01 | 91.12 |
| W1_132 | 97978364 | 97952258 | 14696754600 | 14597152174 | 99.32 | 97.31 | 91.96 |
| W1_133 | 101022724 | 100956802 | 15153408600 | 15040977256 | 99.26 | 97.16 | 91.21 |
| W1_134 | 99106470 | 99040516 | 14865970500 | 14765030688 | 99.32 | 97.54 | 92.21 |
| W1_135 | 97638216 | 97601676 | 14645732400 | 14545098440 | 99.31 | 96.93 | 90.93 |
| W1_136 | 97525482 | 97499632 | 14628822300 | 14512314050 | 99.20 | 97.36 | 91.97 |
| W1_137 | 95705112 | 95675864 | 14355766800 | 14248970912 | 99.26 | 97.17 | 91.59 |
| W1_138 | 109947614 | 109913476 | 16492142100 | 16401609488 | 99.45 | 96.74 | 90.43 |
| W1_140 | 122512278 | 122475508 | 18376841700 | 18247465540 | 99.30 | 97.51 | 92.54 |
| W1_141 | 98756422 | 98688864 | 14813463300 | 14706666518 | 99.28 | 97.71 | 92.77 |
| W1_142 | 107754460 | 107731658 | 16163169000 | 16038655504 | 99.23 | 97.04 | 91.25 |
| W1_144 | 100331610 | 100297854 | 15049741500 | 14922821426 | 99.16 | 97.26 | 91.75 |
| W1_145 | 96534048 | 96505534 | 14480107200 | 14372570818 | 99.26 | 97.18 | 91.65 |
| W1_146 | 125036138 | 124996242 | 18755420700 | 18607389976 | 99.21 | 97.17 | 91.66 |
| W1_148 | 100361742 | 100343738 | 15054261300 | 14933058156 | 99.19 | 97.34 | 91.99 |
| W1_149 | 98648918 | 98623786 | 14797337700 | 14687394028 | 99.26 | 96.85 | 90.52 |
| W1_150 | 112440640 | 112406430 | 16866096000 | 16751125556 | 99.32 | 97.50 | 92.51 |
| W1_151 | 99979536 | 99946372 | 14996930400 | 14881134098 | 99.23 | 96.98 | 90.93 |
| W1_152 | 98490918 | 98463396 | 14773637700 | 14659732496 | 99.23 | 97.20 | 91.56 |
| W1_153 | 100843692 | 100821746 | 15126553800 | 15017349412 | 99.28 | 97.41 | 92.12 |
| W1_154 | 95961338 | 95931846 | 14394200700 | 14300207722 | 99.35 | 96.93 | 90.59 |
| W1_155 | 98729754 | 98707078 | 14809463100 | 14720192566 | 99.40 | 96.84 | 90.52 |
| W1_156 | 117000262 | 116924240 | 17550039300 | 17419333690 | 99.26 | 97.18 | 91.36 |
| W1_157 | 97007104 | 96979480 | 14551065600 | 14405977144 | 99.00 | 97.35 | 91.83 |
| W1_160 | 97598212 | 97528692 | 14639731800 | 14533882936 | 99.28 | 97.29 | 91.65 |
| W1_161 | 103960612 | 103926652 | 15594091800 | 15474023034 | 99.23 | 97.12 | 91.50 |
| W1_162 | 118686596 | 118649970 | 17802989400 | 17666059800 | 99.23 | 97.09 | 91.36 |
| W1_163 | 105337116 | 105312010 | 15800567400 | 15676373344 | 99.21 | 97.34 | 92.06 |
| W1_166 | 96949390 | 96910030 | 14542408500 | 14419448554 | 99.15 | 97.51 | 92.57 |
| W1_167 | 99033420 | 99004726 | 14855013000 | 14747098106 | 99.27 | 97.16 | 91.59 |
| W1_168 | 98151332 | 98088928 | 14722699800 | 14609435004 | 99.23 | 97.65 | 92.61 |
| W1_169 | 99316868 | 99282942 | 14897530200 | 14787976534 | 99.26 | 96.70 | 90.35 |
| W1_171 | 103055408 | 103022534 | 15458311200 | 15355181542 | 99.33 | 97.69 | 93.08 |
| W1_173 | 99774930 | 99749232 | 14966239500 | 14865501572 | 99.33 | 97.25 | 91.67 |
| W1_200 | 122061550 | 122024932 | 18790315385 | 18640238633 | 99.20 | 97.36 | 91.91 |
| W1_201 | 150779044 | 150733810 | 22813310725 | 22530674550 | 98.76 | 97.37 | 91.64 |
| W1_202 | 132738035 | 132698214 | 19845986019 | 19591579670 | 98.72 | 96.31 | 88.98 |
| W1_203 | 191792866 | 191735328 | 28798568618 | 28355626098 | 98.46 | 97.31 | 91.68 |
| W1_204 | 97863479 | 97834120 | 14626987454 | 14446031943 | 98.76 | 98.22 | 93.97 |
| W1_205 | 136205864 | 136165002 | 20438021735 | 20122211973 | 98.45 | 97.35 | 91.74 |
| W1_206 | 120782497 | 120746262 | 18125958325 | 17959718689 | 99.08 | 97.55 | 92.52 |
| W1_207 | 173687710 | 173635604 | 25707182545 | 25312902476 | 98.47 | 95.77 | 87.48 |
| W1_208 | 126234214 | 126196344 | 19124292631 | 18941195707 | 99.04 | 97.62 | 92.31 |
| W1_209 | 145183591 | 145140036 | 21905031983 | 21592220748 | 98.57 | 94.26 | 83.83 |
| W1_210 | 146870515 | 146826454 | 21945170003 | 21631785584 | 98.57 | 94.31 | 83.95 |
| W1_211 | 151037825 | 150992514 | 22861446811 | 22578214273 | 98.76 | 97.39 | 91.66 |
| W1_212 | 140005990 | 139963988 | 21079906703 | 20845457320 | 98.89 | 97.61 | 92.56 |
| W1_213 | 134327288 | 134286990 | 20155210183 | 19931905369 | 98.89 | 95.82 | 87.68 |
| W1_214 | 135867432 | 135826672 | 20300582919 | 20036808175 | 98.70 | 97.73 | 92.54 |
| W1_215 | 177545700 | 177492436 | 25737966211 | 25343214001 | 98.47 | 95.92 | 87.67 |
| W1_216 | 109747754 | 109714830 | 16228108618 | 16064384649 | 98.99 | 98.05 | 93.42 |
| W1_217 | 107982883 | 107950488 | 16190708082 | 16027361444 | 98.99 | 97.98 | 93.29 |
| W1_218 | 132176157 | 132136504 | 19839833764 | 19585506280 | 98.72 | 96.28 | 88.94 |
| W1_219 | 113044265 | 113010352 | 17032722619 | 16843892740 | 98.89 | 95.65 | 87.30 |
| W1_220 | 145135101 | 145091560 | 21904593882 | 21591788904 | 98.57 | 94.26 | 83.84 |
| W1_221 | 172276063 | 172224380 | 25655876436 | 25262383266 | 98.47 | 95.75 | 87.46 |
| W1_222 | 104419062 | 104387736 | 15641712870 | 15520169336 | 99.22 | 97.16 | 91.55 |
| W1_223 | 101102305 | 101071974 | 15165492321 | 15066560721 | 99.35 | 96.85 | 90.39 |
| W1_224 | 113363211 | 113329202 | 17032722619 | 16843892740 | 98.89 | 95.68 | 87.32 |
| W1_225 | 130888843 | 130849576 | 19402262798 | 19153544551 | 98.72 | 96.29 | 88.97 |
| W1_226 | 129625488 | 129586600 | 19687325458 | 19434952984 | 98.72 | 96.21 | 88.92 |
| W1_227 | 111829243 | 111795694 | 16768473440 | 16648959334 | 99.29 | 97.56 | 92.43 |
| W1_228 | 165266770 | 165217190 | 24352852065 | 24038256704 | 98.71 | 97.12 | 91.76 |
| W1_229 | 131025988 | 130986680 | 19639650163 | 19466003602 | 99.12 | 96.30 | 88.98 |
| W1_230 | 107211732 | 107179568 | 16112992683 | 15950430109 | 98.99 | 97.98 | 93.29 |
| W1_231 | 116877857 | 116842794 | 17465799111 | 17337281103 | 99.26 | 97.16 | 91.34 |
| W1_232 | 143511960 | 143468906 | 21405957801 | 21126658592 | 98.70 | 97.18 | 91.09 |
| W1_233 | 95256101 | 95227524 | 14331039041 | 14248542514 | 99.42 | 97.30 | 91.93 |
| W1_234 | 137668699 | 137627398 | 20337682086 | 20023422779 | 98.45 | 97.38 | 91.75 |
| W1_235 | 171080114 | 171028790 | 26201017522 | 25799163332 | 98.47 | 95.77 | 88.98 |
| W1_236 | 98403011 | 98373490 | 14775262800 | 14662664588 | 99.24 | 97.21 | 86.55 |
| W1_237 | 153164847 | 153118898 | 23023421317 | 22738182062 | 98.76 | 97.42 | 91.68 |
| W1_238 | 115473100 | 115438458 | 17217751709 | 16992928774 | 98.69 | 97.71 | 93.11 |
| W1_239 | 154061260 | 154015042 | 23032860920 | 22747504717 | 98.76 | 97.56 | 91.73 |
| W1_240 | 168124641 | 168074204 | 24884779612 | 24563312698 | 98.71 | 97.43 | 91.42 |
| W1_241 | 166848503 | 166798448 | 24398805747 | 24083616747 | 98.71 | 97.29 | 91.19 |
| W1_242 | 146018548 | 145974742 | 21839833187 | 21527953013 | 98.57 | 94.19 | 83.87 |
| W1_243 | 166582305 | 166532330 | 24281691479 | 23968015386 | 98.71 | 96.03 | 90.21 |
| W1_244 | 133327160 | 133287162 | 19857888012 | 19603329091 | 98.72 | 96.48 | 90.91 |
| W1_250 | 113990960 | 113939726 | 17098644000 | 16899378406 | 98.83 | 98.25 | 94.22 |
| W1_251 | 107926182 | 107871110 | 16188927300 | 16024156454 | 98.98 | 97.97 | 93.28 |
| W1_252 | 111802860 | 111755654 | 16770429000 | 16621269806 | 99.11 | 97.67 | 92.34 |
| W1_253 | 110217450 | 110167382 | 16532617500 | 16383472976 | 99.10 | 97.79 | 92.79 |
| W1_254 | 111915854 | 111862552 | 16787378100 | 16558925424 | 98.64 | 97.96 | 93.27 |
| W1_255 | 114680652 | 114609722 | 17202097800 | 16975951432 | 98.69 | 97.94 | 93.23 |
| W1_256 | 97405130 | 97347568 | 14610769500 | 14428716042 | 98.75 | 98.22 | 93.97 |
| W1_257 | 98324342 | 98266148 | 14748651300 | 14527449246 | 98.50 | 97.74 | 92.57 |
| W1_258 | 114612744 | 114563480 | 17191911600 | 17012165214 | 98.95 | 97.99 | 93.42 |
| W1_259 | 135291458 | 135249766 | 20293718700 | 20113194402 | 99.11 | 97.88 | 93.17 |
| W1_260 | 127534820 | 127497378 | 19130223000 | 18945364216 | 99.03 | 97.62 | 92.31 |
| W1_261 | 105969022 | 105917734 | 15895353300 | 15735469348 | 98.99 | 97.84 | 92.97 |

**Table S11.** Statistics of coverage depth and coverage ratio.

| **Sample** | **Alignment rate** | **Average depth**  **(X)** | **Coverage**  **(%)** | **Coverage, 5X**  **(%)** | **Coverage, 10X**  **(%)** |
| --- | --- | --- | --- | --- | --- |
| 22Z001 | 99.64 | 14.06 | 99.44 | 95.21 | 73.80 |
| 22Z005 | 93.70 | 13.65 | 99.30 | 94.24 | 70.94 |
| 22Z008 | 99.83 | 18.00 | 99.48 | 97.48 | 87.02 |
| 22Z009 | 99.72 | 12.43 | 99.34 | 93.34 | 64.60 |
| 22Z010 | 99.75 | 15.53 | 99.49 | 96.56 | 80.55 |
| 22Z018 | 99.75 | 13.98 | 99.42 | 95.31 | 73.86 |
| 22Z021 | 99.75 | 11.17 | 99.23 | 90.25 | 54.09 |
| 22Z022 | 99.78 | 15.94 | 99.46 | 96.65 | 81.76 |
| 22Z024 | 99.78 | 13.72 | 99.39 | 94.94 | 72.58 |
| 22Z025 | 99.74 | 16.08 | 99.44 | 96.82 | 82.78 |
| 22Z029 | 99.72 | 15.63 | 99.50 | 96.61 | 80.88 |
| 22Z031 | 99.75 | 15.56 | 99.46 | 96.55 | 80.84 |
| 22Z032 | 99.77 | 14.67 | 99.46 | 95.86 | 76.83 |
| 22Z040 | 99.39 | 13.41 | 99.36 | 95.46 | 72.62 |
| 22Z043 | 99.73 | 11.86 | 99.21 | 91.58 | 60.59 |
| 22Z047 | 99.71 | 12.34 | 99.31 | 92.76 | 63.48 |
| 22Z058 | 99.75 | 13.46 | 99.34 | 94.29 | 70.71 |
| 22Z061 | 99.76 | 12.32 | 99.25 | 92.60 | 63.75 |
| 22Z063 | 99.73 | 13.52 | 99.42 | 94.94 | 71.74 |
| 22Z085 | 99.72 | 12.76 | 99.29 | 93.31 | 66.83 |
| 22Z086 | 99.73 | 13.37 | 99.29 | 93.87 | 69.75 |
| 22Z036 | 99.74 | 13.60 | 99.37 | 94.37 | 71.08 |
| 22Z088 | 99.30 | 11.74 | 99.23 | 93.46 | 61.76 |
| 22Z091 | 99.46 | 15.18 | 99.33 | 96.72 | 81.69 |
| W1_1 | 99.79 | 11.94 | 99.28 | 92.84 | 61.32 |
| W1_2 | 99.44 | 6.23 | 98.64 | 65.59 | 9.35 |
| W1_3 | 99.73 | 8.51 | 98.58 | 78.97 | 30.36 |
| W1_4 | 99.41 | 5.75 | 98.38 | 59.53 | 6.62 |
| W1_5 | 99.32 | 5.76 | 98.41 | 59.23 | 6.41 |
| W1_6 | 99.77 | 8.96 | 98.75 | 82.06 | 35.20 |
| W1_7 | 99.74 | 7.10 | 98.04 | 70.38 | 19.39 |
| W1_8 | 99.77 | 8.34 | 98.61 | 79.49 | 30.82 |
| W1_9 | 99.74 | 6.80 | 97.78 | 67.77 | 17.48 |
| W1_10 | 99.82 | 7.85 | 98.56 | 76.40 | 25.59 |
| W1_11 | 99.79 | 8.22 | 98.66 | 78.77 | 29.40 |
| W1_12 | 99.72 | 7.07 | 98.44 | 71.75 | 18.95 |
| W1_13 | 99.77 | 11.09 | 99.20 | 90.31 | 55.09 |
| W1_14 | 99.73 | 8.74 | 98.98 | 82.38 | 33.18 |
| W1_16 | 99.41 | 5.87 | 98.25 | 59.88 | 7.11 |
| W1_17 | 99.31 | 5.93 | 98.28 | 61.03 | 7.57 |
| W1_18 | 99.76 | 11.27 | 99.13 | 90.26 | 55.71 |
| W1_19 | 99.58 | 10.08 | 99.16 | 88.10 | 46.12 |
| W1_20 | 99.80 | 9.64 | 99.11 | 86.86 | 42.59 |
| W1_21 | 99.65 | 7.96 | 98.34 | 75.95 | 26.38 |
| W1_22 | 99.72 | 8.02 | 98.50 | 77.27 | 27.33 |
| W1_23 | 99.77 | 6.41 | 97.20 | 62.99 | 14.10 |
| W1_24 | 99.68 | 8.38 | 98.59 | 79.25 | 30.57 |
| W1_25 | 99.79 | 8.42 | 98.59 | 79.80 | 31.73 |
| W1_26 | 99.78 | 8.03 | 98.53 | 77.31 | 27.43 |
| W1_27 | 99.70 | 9.24 | 98.81 | 83.44 | 38.32 |
| W1_28 | 99.80 | 8.82 | 98.66 | 81.37 | 34.98 |
| W1_29 | 99.39 | 5.74 | 98.02 | 57.38 | 6.38 |
| W1_30 | 99.38 | 6.65 | 98.61 | 68.89 | 12.28 |
| W1_32 | 99.43 | 5.89 | 98.46 | 61.01 | 7.05 |
| W1_33 | 99.44 | 6.18 | 98.68 | 64.05 | 8.46 |
| W1_34 | 99.38 | 6.02 | 98.30 | 62.09 | 8.10 |
| W1_35 | 99.45 | 6.02 | 98.52 | 62.61 | 7.83 |
| W1_36 | 99.38 | 7.19 | 98.56 | 73.30 | 17.52 |
| W1_37 | 99.36 | 7.06 | 98.79 | 73.19 | 15.35 |
| W1_38 | 99.42 | 5.84 | 98.45 | 60.11 | 6.80 |
| W1_39 | 99.43 | 6.00 | 98.55 | 62.20 | 7.59 |
| W1_40 | 99.35 | 5.78 | 98.10 | 58.44 | 6.62 |
| W1_41 | 99.29 | 6.35 | 98.75 | 66.89 | 9.79 |
| W1_42 | 99.40 | 6.29 | 98.41 | 65.15 | 9.79 |
| W1_43 | 99.39 | 5.84 | 98.12 | 59.83 | 7.40 |
| W1_45 | 99.41 | 5.68 | 97.98 | 57.11 | 6.23 |
| W1_46 | 99.44 | 6.01 | 98.54 | 62.56 | 7.72 |
| W1_47 | 99.43 | 5.90 | 98.44 | 60.88 | 7.05 |
| W1_48 | 99.41 | 6.05 | 98.22 | 62.12 | 8.12 |
| W1_49 | 99.41 | 6.45 | 98.50 | 66.24 | 10.43 |
| W1_50 | 99.40 | 6.14 | 98.31 | 63.38 | 8.95 |
| W1_51 | 99.50 | 6.82 | 98.89 | 71.66 | 13.04 |
| W1_52 | 99.08 | 5.92 | 98.15 | 60.07 | 7.29 |
| W1_54 | 99.40 | 5.91 | 98.46 | 60.53 | 7.01 |
| W1_55 | 99.41 | 6.00 | 98.50 | 62.42 | 7.67 |
| W1_56 | 99.33 | 5.85 | 98.10 | 60.27 | 7.59 |
| W1_57 | 99.40 | 6.05 | 98.57 | 63.20 | 7.97 |
| W1_58 | 99.40 | 6.35 | 98.67 | 66.47 | 9.73 |
| W1_59 | 99.22 | 5.97 | 98.20 | 60.71 | 7.58 |
| W1_60 | 99.31 | 6.30 | 98.43 | 64.87 | 9.51 |
| W1_61 | 99.39 | 6.38 | 98.49 | 66.17 | 10.23 |
| W1_62 | 99.36 | 5.99 | 98.53 | 61.94 | 7.41 |
| W1_63 | 99.37 | 6.52 | 98.53 | 67.54 | 11.12 |
| W1_64 | 99.41 | 6.82 | 98.88 | 71.84 | 12.92 |
| W1_65 | 99.33 | 6.04 | 98.21 | 62.40 | 8.39 |
| W1_66 | 99.45 | 7.06 | 98.71 | 72.86 | 15.74 |
| W1_67 | 99.46 | 5.85 | 98.40 | 59.94 | 6.76 |
| W1_68 | 99.36 | 5.94 | 98.17 | 60.78 | 7.59 |
| W1_69 | 99.46 | 7.09 | 98.94 | 74.29 | 14.72 |
| W1_70 | 99.36 | 6.48 | 98.48 | 66.72 | 10.78 |
| W1_71 | 99.43 | 6.59 | 98.68 | 69.40 | 11.56 |
| W1_72 | 99.30 | 5.98 | 98.18 | 61.10 | 7.80 |
| W1_75 | 99.38 | 5.71 | 98.07 | 58.02 | 6.51 |
| W1_76 | 99.48 | 8.35 | 99.03 | 82.62 | 27.94 |
| W1_78 | 99.31 | 5.74 | 98.00 | 57.88 | 6.46 |
| W1_79 | 99.36 | 5.84 | 98.15 | 59.53 | 7.03 |
| W1_80 | 99.39 | 6.12 | 98.27 | 62.87 | 8.59 |
| W1_81 | 99.09 | 5.84 | 98.04 | 59.17 | 7.08 |
| W1_83 | 99.39 | 6.21 | 98.34 | 63.01 | 8.75 |
| W1_84 | 99.37 | 6.01 | 98.23 | 61.96 | 8.30 |
| W1_85 | 99.39 | 7.43 | 98.86 | 76.13 | 18.21 |
| W1_86 | 99.42 | 5.94 | 98.54 | 61.35 | 7.18 |
| W1_87 | 99.36 | 6.17 | 98.32 | 63.54 | 8.71 |
| W1_88 | 99.35 | 5.75 | 98.06 | 58.55 | 6.75 |
| W1_89 | 99.34 | 5.99 | 98.29 | 61.45 | 7.89 |
| W1_90 | 99.40 | 5.66 | 97.81 | 56.67 | 6.40 |
| W1_91 | 99.38 | 6.03 | 98.31 | 62.26 | 8.16 |
| W1_92 | 99.43 | 7.01 | 98.93 | 74.01 | 14.73 |
| W1_93 | 99.37 | 6.00 | 98.51 | 62.17 | 7.65 |
| W1_94 | 99.22 | 5.75 | 98.08 | 58.17 | 6.47 |
| W1_95 | 99.38 | 6.00 | 98.53 | 62.49 | 7.71 |
| W1_96 | 99.40 | 6.07 | 98.56 | 62.80 | 7.93 |
| W1_97 | 99.41 | 5.94 | 98.24 | 61.12 | 7.70 |
| W1_98 | 99.45 | 7.01 | 98.95 | 73.71 | 14.48 |
| W1_99 | 99.41 | 5.89 | 98.48 | 60.93 | 6.99 |
| W1_100 | 99.36 | 5.87 | 98.15 | 59.63 | 7.07 |
| W1_101 | 99.39 | 6.00 | 98.24 | 61.37 | 7.88 |
| W1_102 | 99.39 | 5.71 | 98.06 | 57.69 | 6.36 |
| W1_103 | 99.19 | 5.98 | 98.47 | 61.70 | 7.35 |
| W1_104 | 99.38 | 6.45 | 98.54 | 66.65 | 10.46 |
| W1_105 | 99.48 | 6.83 | 98.90 | 72.01 | 13.24 |
| W1_106 | 99.36 | 5.70 | 98.03 | 57.49 | 6.27 |
| W1_107 | 99.30 | 5.92 | 98.18 | 60.50 | 7.51 |
| W1_108 | 99.37 | 6.32 | 98.62 | 66.26 | 9.58 |
| W1_109 | 99.36 | 7.34 | 98.87 | 75.62 | 18.03 |
| W1_110 | 99.34 | 5.98 | 98.21 | 60.96 | 7.72 |
| W1_112 | 99.48 | 5.86 | 98.47 | 60.14 | 6.73 |
| W1_113 | 99.41 | 5.90 | 98.46 | 60.45 | 6.89 |
| W1_114 | 99.34 | 6.10 | 98.56 | 63.05 | 8.06 |
| W1_115 | 99.35 | 6.18 | 98.40 | 63.63 | 8.91 |
| W1_116 | 99.41 | 7.13 | 98.83 | 73.71 | 15.68 |
| W1_117 | 99.33 | 5.96 | 98.24 | 60.70 | 7.48 |
| W1_118 | 99.45 | 7.98 | 99.19 | 81.83 | 22.98 |
| W1_119 | 99.37 | 6.72 | 98.64 | 70.05 | 12.89 |
| W1_120 | 99.40 | 6.23 | 98.67 | 65.33 | 9.13 |
| W1_121 | 99.43 | 6.19 | 98.42 | 64.05 | 9.07 |
| W1_122 | 99.41 | 5.84 | 98.16 | 59.68 | 7.10 |
| W1_123 | 99.40 | 5.86 | 98.19 | 59.95 | 7.20 |
| W1_124 | 99.46 | 7.12 | 98.75 | 73.06 | 16.26 |
| W1_125 | 99.36 | 6.08 | 98.30 | 62.52 | 8.39 |
| W1_126 | 99.40 | 6.06 | 98.37 | 62.88 | 8.45 |
| W1_127 | 99.42 | 6.86 | 98.60 | 71.08 | 13.63 |
| W1_128 | 99.38 | 6.02 | 98.52 | 62.96 | 7.92 |
| W1_129 | 99.31 | 6.03 | 98.54 | 63.37 | 8.07 |
| W1_130 | 99.20 | 5.95 | 98.24 | 60.82 | 7.54 |
| W1_131 | 99.33 | 6.03 | 98.50 | 62.66 | 7.88 |
| W1_132 | 99.41 | 5.86 | 98.19 | 59.75 | 7.03 |
| W1_133 | 99.21 | 6.00 | 98.25 | 61.55 | 7.90 |
| W1_134 | 99.34 | 5.92 | 98.16 | 60.43 | 7.46 |
| W1_135 | 99.41 | 5.83 | 98.18 | 59.26 | 6.81 |
| W1_136 | 99.36 | 5.81 | 98.45 | 59.71 | 6.52 |
| W1_137 | 99.42 | 5.70 | 97.96 | 57.14 | 6.09 |
| W1_138 | 99.41 | 6.60 | 98.66 | 69.05 | 11.86 |
| W1_140 | 99.41 | 7.30 | 98.91 | 75.24 | 17.12 |
| W1_141 | 98.43 | 5.82 | 98.39 | 59.68 | 6.64 |
| W1_142 | 99.41 | 6.43 | 98.55 | 66.84 | 10.60 |
| W1_144 | 99.35 | 5.96 | 98.50 | 61.36 | 7.23 |
| W1_145 | 99.39 | 5.76 | 98.08 | 58.07 | 6.57 |
| W1_146 | 99.43 | 7.45 | 99.08 | 77.59 | 18.05 |
| W1_148 | 99.28 | 5.97 | 98.46 | 61.33 | 7.35 |
| W1_149 | 99.29 | 5.90 | 98.49 | 60.97 | 7.10 |
| W1_150 | 99.42 | 6.72 | 98.68 | 69.99 | 12.68 |
| W1_151 | 99.35 | 5.95 | 98.53 | 61.43 | 7.15 |
| W1_152 | 99.42 | 5.88 | 98.53 | 60.76 | 7.02 |
| W1_153 | 99.47 | 6.04 | 98.55 | 62.87 | 7.87 |
| W1_154 | 99.22 | 5.73 | 98.03 | 58.11 | 6.67 |
| W1_155 | 99.41 | 5.93 | 98.38 | 61.36 | 7.23 |
| W1_156 | 99.33 | 6.98 | 98.93 | 73.56 | 14.29 |
| W1_157 | 98.99 | 5.72 | 98.34 | 58.35 | 6.14 |
| W1_160 | 99.30 | 5.83 | 98.32 | 60.09 | 6.70 |
| W1_161 | 99.40 | 6.19 | 98.44 | 64.08 | 9.10 |
| W1_162 | 99.43 | 7.07 | 99.01 | 74.25 | 14.62 |
| W1_163 | 99.42 | 6.25 | 98.67 | 64.61 | 8.64 |
| W1_166 | 99.45 | 5.77 | 98.13 | 58.13 | 6.53 |
| W1_167 | 99.43 | 5.91 | 98.20 | 60.15 | 7.30 |
| W1_168 | 99.39 | 5.87 | 98.44 | 60.64 | 7.12 |
| W1_169 | 99.46 | 5.92 | 98.50 | 60.71 | 6.95 |
| W1_171 | 99.38 | 6.16 | 98.39 | 63.85 | 8.95 |
| W1_173 | 99.38 | 5.96 | 98.50 | 61.48 | 7.26 |
| W1_200 | 92.97 | 6.49 | 98.05 | 63.54 | 11.85 |
| W1_201 | 98.00 | 8.76 | 99.06 | 84.73 | 31.23 |
| W1_202 | 99.34 | 7.84 | 99.08 | 79.80 | 21.64 |
| W1_203 | 98.68 | 11.25 | 99.38 | 93.90 | 56.60 |
| W1_204 | 98.91 | 5.73 | 97.67 | 56.74 | 7.45 |
| W1_205 | 98.87 | 8.02 | 98.95 | 79.74 | 23.86 |
| W1_206 | 99.50 | 7.19 | 98.75 | 73.73 | 16.52 |
| W1_207 | 92.44 | 9.32 | 99.26 | 87.39 | 36.95 |
| W1_208 | 99.43 | 7.60 | 98.89 | 77.73 | 21.66 |
| W1_209 | 99.53 | 8.73 | 99.27 | 86.59 | 31.95 |
| W1_210 | 99.45 | 8.77 | 99.27 | 86.55 | 32.15 |
| W1_211 | 99.53 | 9.09 | 99.33 | 88.48 | 35.77 |
| W1_212 | 99.48 | 8.44 | 99.22 | 85.08 | 28.73 |
| W1_213 | 99.41 | 8.04 | 99.03 | 81.03 | 24.65 |
| W1_214 | 99.49 | 8.19 | 99.21 | 83.67 | 26.40 |
| W1_215 | 99.44 | 10.66 | 99.34 | 91.95 | 52.45 |
| W1_216 | 99.44 | 6.59 | 98.46 | 68.34 | 12.47 |
| W1_217 | 99.45 | 6.49 | 98.52 | 67.30 | 11.40 |
| W1_218 | 99.44 | 7.93 | 99.05 | 80.19 | 23.59 |
| W1_219 | 99.43 | 6.83 | 98.72 | 71.82 | 14.39 |
| W1_220 | 99.44 | 8.69 | 99.14 | 84.64 | 31.44 |
| W1_221 | 99.50 | 10.36 | 99.34 | 92.61 | 49.52 |
| W1_222 | 99.45 | 6.30 | 98.67 | 66.64 | 10.05 |
| W1_223 | 99.52 | 6.12 | 98.56 | 65.12 | 9.04 |
| W1_224 | 99.17 | 6.72 | 98.78 | 69.70 | 12.80 |
| W1_225 | 99.48 | 7.76 | 99.06 | 79.47 | 20.74 |
| W1_226 | 99.50 | 7.71 | 99.11 | 79.38 | 20.35 |
| W1_227 | 99.46 | 6.74 | 98.58 | 70.35 | 13.48 |
| W1_228 | 99.45 | 9.94 | 99.27 | 90.00 | 45.35 |
| W1_229 | 99.40 | 7.85 | 99.14 | 81.05 | 22.09 |
| W1_230 | 99.47 | 6.48 | 98.78 | 68.87 | 11.53 |
| W1_231 | 99.38 | 7.02 | 98.69 | 73.24 | 15.80 |
| W1_232 | 99.47 | 8.52 | 99.21 | 84.80 | 27.91 |
| W1_233 | 99.51 | 5.68 | 98.23 | 57.19 | 6.01 |
| W1_234 | 99.42 | 8.21 | 99.04 | 82.00 | 26.09 |
| W1_235 | 99.49 | 10.21 | 99.35 | 92.14 | 46.97 |
| W1_236 | 99.47 | 5.91 | 98.18 | 60.80 | 7.83 |
| W1_237 | 99.45 | 9.12 | 99.17 | 86.70 | 35.62 |
| W1_238 | 99.49 | 6.88 | 98.61 | 71.19 | 14.22 |
| W1_239 | 99.45 | 9.19 | 99.18 | 87.15 | 36.22 |
| W1_240 | 99.49 | 10.04 | 99.28 | 90.16 | 45.33 |
| W1_241 | 99.52 | 9.90 | 99.16 | 89.10 | 43.44 |
| W1_242 | 99.46 | 8.68 | 99.12 | 84.49 | 30.46 |
| W1_243 | 99.48 | 9.89 | 99.21 | 89.36 | 43.29 |
| W1_244 | 99.42 | 7.93 | 99.13 | 81.27 | 22.30 |
| W1_250 | 99.49 | 6.81 | 98.59 | 70.67 | 14.71 |
| W1_251 | 99.44 | 6.43 | 98.30 | 65.81 | 11.75 |
| W1_252 | 99.50 | 6.71 | 98.60 | 70.21 | 13.63 |
| W1_253 | 99.50 | 6.62 | 98.70 | 69.33 | 13.03 |
| W1_254 | 99.47 | 6.62 | 98.64 | 68.27 | 12.73 |
| W1_255 | 99.43 | 6.79 | 98.66 | 69.99 | 14.19 |
| W1_256 | 99.43 | 5.78 | 97.75 | 57.80 | 7.73 |
| W1_257 | 99.48 | 5.81 | 97.72 | 57.80 | 8.04 |
| W1_258 | 99.45 | 6.84 | 98.52 | 70.11 | 15.13 |
| W1_259 | 99.50 | 8.09 | 98.98 | 80.66 | 26.15 |
| W1_260 | 95.12 | 7.20 | 98.89 | 73.93 | 16.24 |
| W1_261 | 99.45 | 6.32 | 98.14 | 64.72 | 11.41 |

**Table S12.** Suggested loci in formation of speed traits.

| **Chromosome Position** | **Model** | ***P*-value** | **Gene** | **Significance** |
| --- | --- | --- | --- | --- |
| 1:67405231 | FarmCPU | 9.20×10^-8^ | LOC100630769 | Suggestive |
| 8:1999945 | Blink | 1.20×10^-8^ | LOC100056031 | Suggestive |
| 16:63926989 | Blink | 2.96×10^-8^ | EFHB | Suggestive |
| 25:18242507 | Blink | 8.69×10^-8^ | LOC100034197, LOC100056127,  LOC111770708, ZFP37 | Suggestive |

**Table S13.** Suggested loci in formation of ranking score traits.

| **Chromosome Position** | **Model** | ***P*-value** | **Gene** | **Significance** |
| --- | --- | --- | --- | --- |
| 6:49636713 | FarmCPU | 7.23×10^-10^ | ETNK1 | Significant |
| 6:80947162 | Blink, FarmCPU | 9.42×10^-11^ 1.87×10^-12^ | RXYLT1, SRGAP1 | Significant |
| 1:157101968 | FarmCPU | 3.34×10^-9^ | LOC100058263, LOC100072092, LOC100072099, LOC100072107, LOC100072110, LOC100072112, LOC100072117, LOC100072120, LOC100072129, LOC100072136, LOC111772402, LOC111775721 | Suggestive |
| 1:12254537 | FarmCPU | 3.74×10^-8^ | PLPP4 | Suggestive |
| 4:59313328 | Blink | 7.05×10^-9^ | JAZF1 | Suggestive |
| 4:102299918 | Blink | 6.01×10^-8^ | GIMAP8, LOC100054458, LOC100063113, LOC100146250, LOC100146699, LOC102150984, LOC111773116, LOC111773285, REPIN1, ZNF775 | Suggestive |
| 6:50947912 | FarmCPU | 1.64×10^-8^ | SOX5 | Suggestive |
| 6:38925972 | FarmCPU | 5.36×10^-8^ | LOC100062904, LOC100630523, LOC102149853, LOC106783275 | Suggestive |
| 8:5827539 | FarmCPU | 7.58×10^-8^ | LOC100060228, LOC102147971, LOC111774596 | Suggestive |
| 8:33324963 | FarmCPU | 1.98×10^-8^ | ANHX(dist=542735), LOC111774691(dist=15386) | Suggestive |
| 12:7585451 | FarmCPU | 1.00×10^-8^ | LOC111775890, LOC111775891 | Suggestive |
| 12:26158716 | FarmCPU | 1.42×10^-8^ | AHNAK, LOC100146450, LOC100629936 | Suggestive |
| 12:29506645 | FarmCPU | 2.12×10^-8^ | AP5B1, EHBP1L1, FAM89B, KAT5, KCNK7, LTBP3, MAP3K11, OVOL1, PCNX3, RELA, RNASEH2C, SCYL1, SIPA1, SSSCA1 | Suggestive |
| 23:49023850 | FarmCPU | 2.82×10^-8^ | ACO1, DDX58, NDUFB6, TOPORS | Suggestive |
| 23:9293033 | FarmCPU | 3.40×10^-8^ | LOC100061516(dist=231435), LOC100061947(dist=230490) | Suggestive |
| 23:40022833 | FarmCPU | 5.81×10^-8^ | IFNA1, IFND1, IFND2, IFNE, LOC100053069, LOC100053110, LOC100053210, LOC100053263, LOC100629951, LOC111770143, LOC111770185 | Suggestive |
